# Atmospheric air plasma pre-activation and customizable covalent functionalization of PVDF-membranes of microtiter filter plates

**DOI:** 10.1038/s41598-024-85040-5

**Published:** 2025-01-25

**Authors:** Bálint Árpád Ádám, Sára Spátay, Bálint Jávor, Szabolcs László, Levente Illés, Péter Fürjes, Tünde Tóth, Péter Huszthy, Ádám Golcs

**Affiliations:** 1 Department of Organic Chemistry and Technology, Budapest University of Technology and Economics, Szent Gellért Tér 4, 1111 Budapest, Hungary; 2 Department of Pharmaceutical Chemistry, Semmelweis University, Hőgyes Endre U. 9, 1092 Budapest, Hungary; 3 Department of Inorganic and Analytical Chemistry, Budapest University of Technology and Economics, Szent Gellért Tér 4, 1111 Budapest, Hungary; 4 HUN-REN, Computation-Driven Chemistry Research Group, Műegyetem Rkp. 3, 1111 Budapest, Hungary; 5 Institute of Technical Physics and Materials Science, HUN-REN Centre for Energy Research, Konkoly-Thege Miklós U. 29-33, 1121 Budapest, Hungary; 6 HUN-REN Centre for Energy Research, Konkoly-Thege Miklós U. 29-33, 1121 Budapest, Hungary; 7 Center for Pharmacology and Drug Research & Development, Semmelweis University, Üllői U. 26, 1085 Budapest, Hungary

**Keywords:** PVDF, Membrane, Plasma, Surface polarization, Covalent immobilization, Chemistry, Analytical chemistry, Chemical engineering, Green chemistry, Materials chemistry, Medicinal chemistry, Organic chemistry, Polymer chemistry, Surface chemistry, Engineering, Chemical engineering, Materials science, Materials for devices

## Abstract

Microtiter-plate-based systems are unified platforms of high-throughput experimentation (HTE). These polymeric devices are used worldwide on a daily basis—mainly in the pharmaceutical industry—for parallel syntheses, reaction optimization, various preclinical studies and high-throughput screening methods. Accordingly, laboratory automation today aims to handle these commercially available multiwell plates, making developments focused on their modifications a priority area of modern applied research. We performed the covalent functionalization of the porous PVDF-membrane of microtiter filter plates as the essence of conventional and common sandwich plate systems by introducing a generalizable method. After surface-activation of the indifferent membrane polymer, customizable functionalization becomes feasible by covalently attached monofunctional molecular linkers. The study was designed with future adaptability, and thus, industrially widespread atmospheric plasma and two different chemical treatments were investigated and compared in terms of practical implementation, polarization effects, extent of labeling, effects on morphology and porosity as well as on permeability. For critical comparison, contact angle measurements, surface ATR-FTIR, ^1^H-NMR, ^19^F-NMR, UV–Vis spectroscopy, scanning electron microscopy and permeability tests were used.

## Introduction

Microtiter plates are essential tools for high-throughput experimentation, playing an indispensable role in diagnostic applications, biotechnology, parallel organic synthesis and high-throughput screening methods. This is mainly due to their high capacity for parallel experiments and their ease of automation by applying commercially available plates of the same size and uniform properties. Therefore, studies on exploring innovative uses of such microplates are desirable and relevant research topics.

**Fig. 1:**
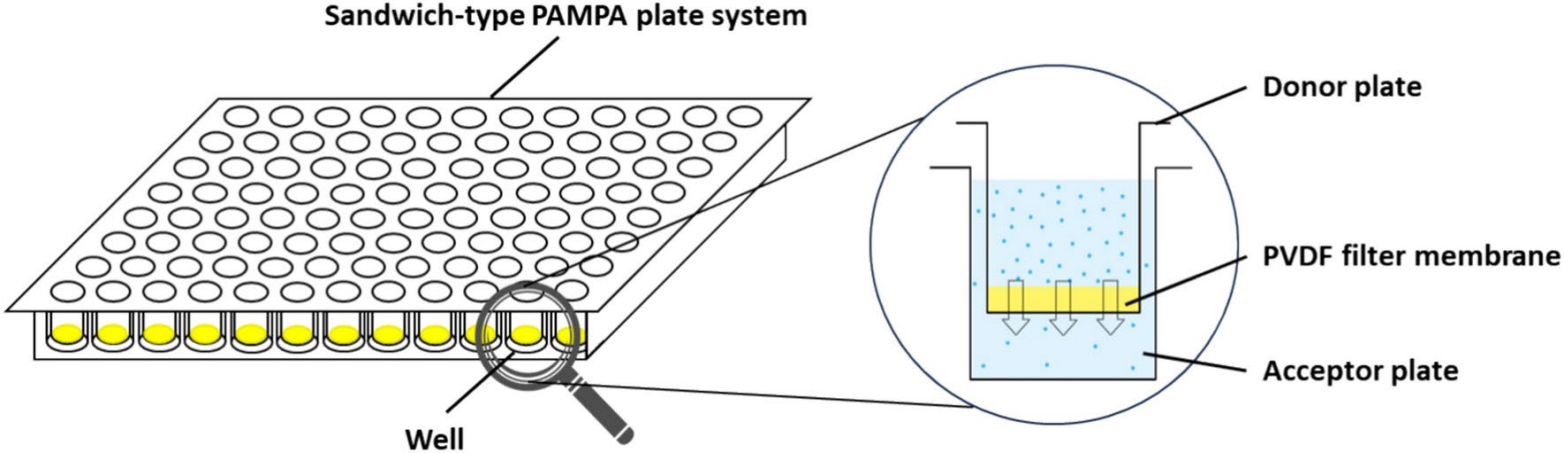
Sandwich-type PAMPA plate system with poly(vinylidene fluoride) (PVDF) filter membrane.

One commonly used type of microtiter plate is the filter plate, which typically has an inert porous filter membrane at the bottom of each well. High-throughput filtrations can be performed with this device, and the time required for the filtration can optionally be reduced using vacuum manifolds^1,2^. When the wells are filled with solid stationary phase, high-throughput process development (HTPD) can be performed to simulate the performance of chromatography columns in miniaturized form with negligible material input^3–6^. In addition, filter plates can also be used for filter-aided sample preparation (FASP)^7,8^ and enzyme-linked immunospot assay (ELISPOT)^9–11^. Similar plate systems form the basis of parallel artificial membrane permeability assay (PAMPA), which models the passive transport of drugs in vitro^12,13^. In this case, the membrane-bottomed filter plate is immersed in the receiver plate (acceptor side of the membrane) creating a sandwich-type system (Fig. 1).

The wells of the filter plate contain a solution of the active pharmaceutical ingredient, and the acceptor side initially contains a solute-free solvent. These two phases are separated by the membrane of the filter plate containing hydrophobic liquid, which mimic biological membranes.

By chemically functionalizing these membranes, alternative uses of microtiter plates are possible. For example, by attaching molecular recognition-capable agents to the membrane, the distribution of chemicals could be influenced, expanding the possibilities of bioassays, various sensing platforms or separation methods. In addition, by covalently attaching catalysts to the surface, heterogeneous catalytic test reactions could be carried out on a miniaturized scale. Alternatively, grafting enzymes onto the membrane would allow the use of these simple tools to study receptor binding or biomimetic syntheses.

One of the most commonly used membrane materials is PVDF, because it has outstanding properties, such as high mechanical strength, chemical resistance, thermal stability (melting point of 140–170 °C; 1% mass loss at 375 °C) and aging resistance^14^. Studies have also shown that PVDF has a high resistance to harsh substances, such as acids, oxidants and different organic solvents^15,16^. Nevertheless, alkalis can cause discoloration and degradation of PVDF^17,18^. Although PVDF has such excellent physical and chemical properties, this can be a disadvantage in certain applications. PVDF lacks reactive functional groups, making it inert to chemical reaction, which can be a limitation in terms of surface functionalization. In order to bind molecules to the membrane, the inert PVDF surface must first be activated. The purpose of the pre-activation is to increase the reactivity of the surface, which allows the further functionalization of the polymer membrane. According to this scheme, various agents can be immobilized to the PVDF membranes, such as enzymes^19–21^, graphene oxide quantum dots^22^, metallic catalyst nanoparticles^23–25^.

Surface modification of PVDF membranes can be divided into two broad categories based on the interactions between the membrane and the modifying agent: physical or chemical modification^26^. Physical modification involves physical interactions between the modifier and the PVDF, leaving the polymer surface chemically unchanged. Such commonly used modifying agents are usually polymers, e.g. polyvinyl alcohol (PVA)^27^, chitosan^28^, poly(ether-block-amide) (PEBAX)^29^ and polydopamine^30^. These coatings are usually prepared by either depositing the modifying polymer directly on the membrane or by immersing it in the monomer solution and then initializing the polymerization^26^. Therefore, physical modifications may also require chemical reactions, but the PVDF membrane is not chemically involved. In contrast, applying chemical modification, reactive groups are formed on the membrane surface to which the modifying agents are covalently attached. The covalent modification naturally results in a chemically more stable surface, allowing multiple uses of the same membrane. Chemical modifications typically consist of two steps: first, increasing the hydrophilicity of the polymer surface by introducing polar anchoring groups, and second, binding modifying agents irreversibly. Chemical surface modifications of PVDF include immersion in a treatment solution (e. g. alkaline activation)^31^, ozone treatment^32^, plasma treatment^33^, electron beam irradiation^34^, etc. These modifications create reactive groups on the surface, which allow additional molecules to be attached. *Gao* et al. produced hydroxyl groups on the membrane surface by oxidation–reduction with KOH/KMnO_4_ and NaHSO_3_/H_2_SO_4_ solutions^35^. In order to increase the hydrophilicity, glycerol was further grafted onto hydroxylated surface. Other researchers have also reported hydroxylated PVDF surfaces using alkaline solutions^31,36^. In these cases, organosilane derivatives were attached to the *O*-containing groups to increase hydrophobicity. To avoid degradation caused by alkaline treatment, *Al-Gharabli* et al. prepared surface hydroxylated membranes using acidic piranha solution^37^. The pre-activated membrane was functionalized with (3-aminopropyl)triethoxysilane creating amine groups on the surface. 1,5-Diamino-2-methylpentane can also be used in basic conditions to graft amino groups to the surface^19^. Another widely used treatment for surface activation is the defluorination-sulfonation method^38–41^.

In contrast, plasma treatment does not require the use of wet chemistry. It has the advantage of being solvent-free and having low energy cost and that it can be used at room temperature as well^42^. Plasma modification is particularly suitable for surface activation, as it treats only the topmost layer of the membranes while leaving the bulk properties unchanged^43^. *Duca* et al. were the first to apply Ar plasma treatment on non-porous PVDF polymer^33^. XPS investigation showed that the surface of PVDF was chemically modified and the *F*/*C* ratio decreased because of *C*–*H*; *C*–*F* bond ruptures, chain scission and double bond formation. Water contact angle (WCA), a measure of wettability, decreased after plasma treatment. Besides Ar plasma treatment, oxygen plasma treatment is also suitable for grafting oxygen-containing functional groups, such as carbonyl, hydroxyl, carboxyl, etc. onto the polymer surface^44^. During the process, ions, radicals and electrons present in the plasma can react with the polymer surface, typically by a radical mechanism, leading to the scission of *C*–*H* and *C*–*F* bonds and the formation of polar functional groups^45^. *Park* and *Inagaki* compared the effects of argon, hydrogen and oxygen plasmas on a PVDF sheet^46^. During the modification, dehydrofluorination and oxidation reactions took place simultaneously. They concluded that hydrogen plasma was the most effective in improving hydrophilicity. It is also reported that the combined use of Ar and O_2_ gas plasma is particularly beneficial for enhancing wetting, as Ar plasma is effective in defluorination, whereas O_2_ plasma is effective in forming *O*-containing functional groups^47^. On the contrary, *Lin* et al. applied CH_4_ plasma on a porous PVDF membrane and opposite effects were experienced compared to the previously mentioned gas plasmas: the WCA and the *F*/*C* ratio increased as the result of CH_4_ plasma modification^42^. The study also showed that the pore size distribution did not change significantly. Vacuum oxygen plasma treatment was also investigated on PVDF membrane sheets^48^. As the result of plasma treatment, the surface was grafted with hydroxyl groups and further functionalized by organosilane derivatives. *Akashi* et al. covalently attached albumin proteins to PVDF membranes pre-activated with atmospheric pressure Ar plasma, extending their biotechnological application^49^.

In this research, we aimed to functionalize the surface of commercially available PVDF filter membranes. The fibrous, porous filter membranes of microplates were activated by atmospheric pressure air plasma, as it does not require specific conditions, such as vacuum or single-component gas supply, and therefore has lower operating costs and greater relevance for industrial applicability than other types of plasmas. The effect of this plasma treatment was also compared with two earlier reported wet-chemical surface treatments. The general method described in this paper enables rapid and efficient surface functionalization of commercially available standard PVDF microtiter filter plates, making possible various molecules to graft on the polymer surface. The present study also aims to facilitate the future development of easy-to-handle, low-cost and reusable microplate-based devices for HTE.

## Results and discussion

### Optimization of atmospheric plasma treatment

The PVDF membranes of the filter plates were activated with atmospheric air plasma. It is worth mentioning that atmospheric pressure plasma was introduced here for the first time to polarize widely applied porous PVDF materials. The effect of the process parameters was investigated by a statistic-based matrix experimental design. The distance of the plasma head from the membrane and the treatment time were independent variables of a 3^2^ full factorial experimental design, whereas the output parameter was the water contact angle of the membrane surface, as a measure of wettability.

**Fig. 2:**
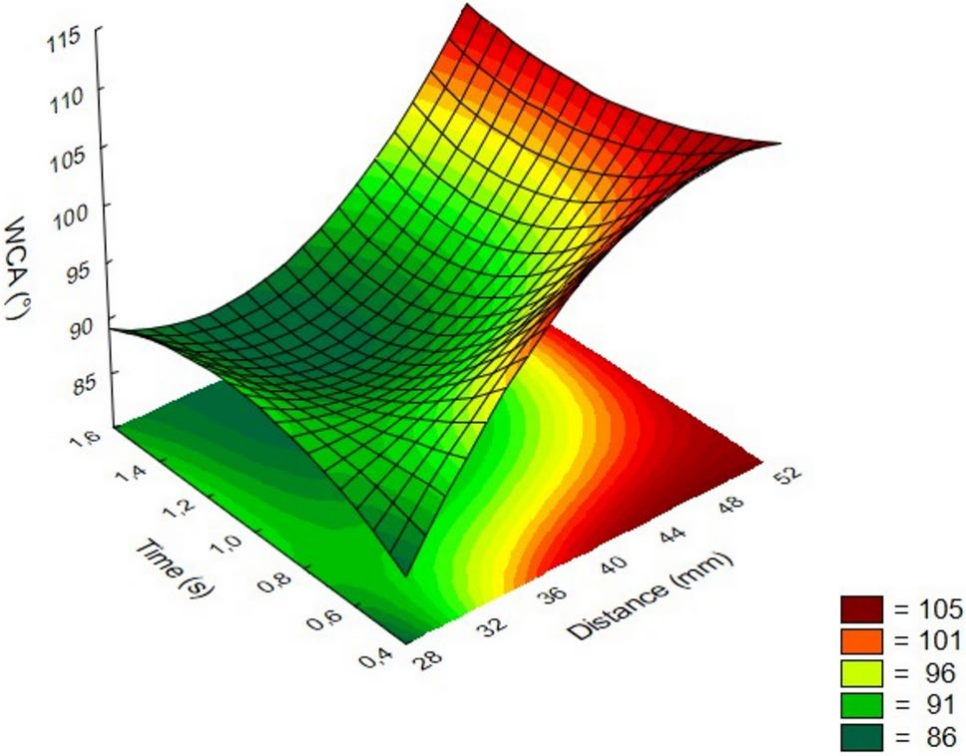
Effects of parameters on the water contact angle.

The WCA as the function of the distance and the time of the treatment is plotted on a response surface diagram (Fig. 2). The analysis of variance (ANOVA) calculations showed that both factors have a great effect on WCA at a significance level of 95%.

It can be seen that the parameters have opposite effects. As the treatment time increases and the treatment distance decreases, the water contact angle decreases, which means a higher hydrophilicity (polarity) of the membrane surface. Nevertheless, the distance has a greater impact on the output parameter. At a distance of 50 mm, the change of WCA is almost negligible, whereas at 30 mm, the time has no significant effect in the examined range. Below 30 mm, the time has a reverse trend, but this is not meaningful, as the membrane melts and ruptures below this critical distance. In summary, the highest achievable hydrophilicity can be characterized by a WCA of 86° under optimal conditions, i.e. 36 mm distance and 1.4 s treatment time. It is worth emphasizing that the latter time of treatment represents an unprecedented fast polarization procedure for PVDF, considering all other reported methods. Subsequently, plasma-treated plate membranes were activated using these optimal conditions.

In order to determine the aging, water contact angles were determined at 0, 12 and 24 h after treatment. In general, an increase of 5° was observed after 24 h, which may be unfavorable for further functionalization. To avoid this negative effect, further manipulation of the membranes was always performed immediately after pre-activation.

To assess the long-term stability of the treatment, additional WCA measurements were performed 1 week and 1 year after plasma activation (see Figure S2 in Supplementary Material). Even after 1 year, only a small increase of 8° in WCA was observed, so the membrane retains its polarized characteristics over long term after treatment by atmospheric pressure air plasma.

### Comparison of wet-chemical and plasma activation of PVDF membrane

The effect of plasma treatment was compared with two reported conventional wet-chemical PVDF-activation methods^31,37^. The exact procedures are detailed in the Experimental Section. The first method was an alkaline treatment with 5% NaOH for 30 min (hereafter referred to as ‘NaOH’), whereas the other was an acidic piranha treatment with a 1:3 mixture of 30 wt% H_2_O_2_ and concentrated H_2_SO_4_ for 10 min after pre-wetting with methanol (hereafter referred to as ‘Pir’). The results of the WCA measurements are shown in Table 1.

**Table 1:**
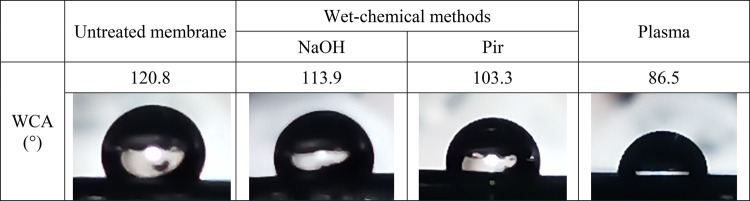
Water contact angles of untreated and differently activated PVDF membranes.

As the results show, the highest hydrophilicity of the membrane is achieved by optimized plasma treatment, whereas the least effective method is alkaline activation. This is due to insufficient interaction between the aqueous alkaline solution and the hydrophobic membrane. In the case of piranha method, interaction is facilitated by methanol prewetting. However, in NaOH treatment, pre-wetting is not applicable, as the methanol in an alkaline environment can do side-reactions by introducing methoxy groups to the surface, which cause problems for further covalent functionalization.

### Surface characterization of activated PVDF membrane

#### Surface ATR-FTIR measurements

**Fig. 3:**
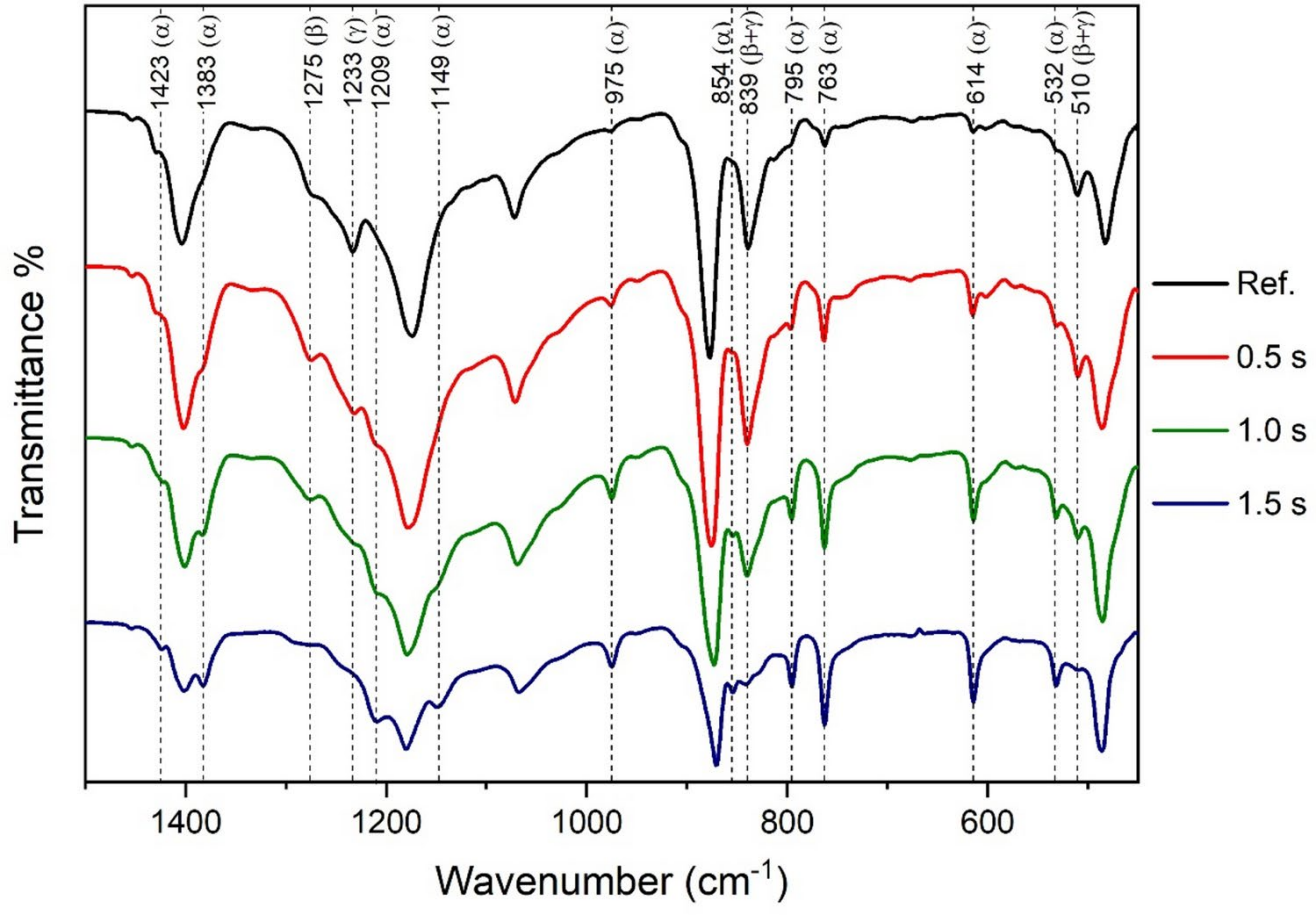
IR spectra of plasma treated (distance = 40 mm). PVDF membranes ($\overline{\nu }$ = 450–1500 cm^−1^) as a function of treatment time.

The modified surface of the plasma treated PVDF membrane was characterized by Fourier transform infrared spectroscopy (FTIR) in attenuated total reflectance mode (ATR). The IR spectra from 450 cm^−1^ to 1500 cm^−1^ of the original and the plasma treated PVDF membranes at a distance of 40 mm and at different treatment times are plotted in Fig. 3.

Before spectra analysis, it is essential to mention that the PVDF polymer has six different polymorphs, namely, α, β, γ, δ, ε and ζ based on the conformation of the polymer chains and their relative positions to each other^50,51^. The ζ phase has not yet been experimentally characterized, but density functional theory (DFT) study has predicted its existence^52^. Schematic models of PVDF polymorphs are presented in the Supplementary Material.

**Fig. 4:**
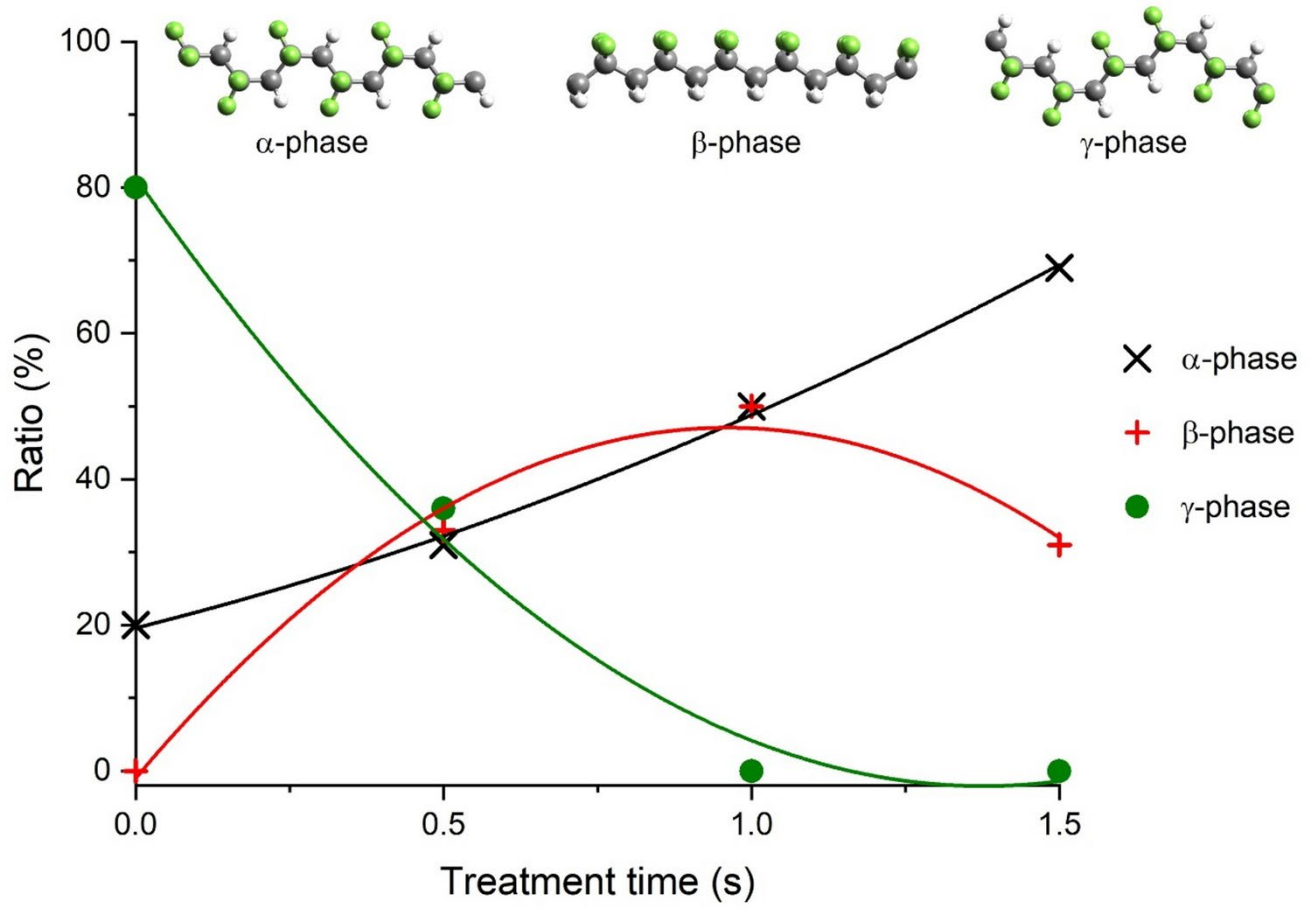
Effect of plasma treatment duration on polymorphic states of PVDF membrane (Colores of atoms in the polymer chains: *C*: grey; *H*: white; *F*: green.)

The polymorphs of PVDF can be distinguished by FTIR^53^. Each phase has uniform vibrational wavenumbers (not marked in Fig. 3), such as 1398–1404; 1171–1182; 1067–1071; and 876–885 cm^−1^. Several peaks (1423; 1383; 1149; 975; 795; 763; 614; and 532 cm^−1^) indicate solely α phase, whereas 837–841 and 508–512 cm^−1^ can be assigned to electroactive polymorphs β and γ together, and 1275; 1233 cm^−1^ peaks are characteristic only for the β and γ, respectively. The proportion of α, β and γ phases of PVDF can be calculated using Eqs. (1), (2a) and (2b) reported in the Experimental Section^53,54^. The crystalline fraction ratios of reference, 0.5 s, 1.0 s and 1.5 s treated PVDF filters are shown in Fig. 4.

Initially, untreated PVDF membrane contains only γ and α phases in 80–20%, respectively. However, as the duration of plasma treatment increases, the absorbance of α peaks (e.g., 614; 763 cm^−1^) are becoming greater while γ peak (1233 cm^−1^) disappears. If the treatment lasts longer than one second, no polymorph γ can be observed in the membrane. Besides, the treatment initially increases fraction β, then further treatment causes this phase to decrease, giving a maximum with 50% at about one second. Changes of the same character can be observed when the treatment time is a fixed parameter, and the distance is the variable. Previous studies showed that plasma treatment increased the proportion of β phase and decreased that of α, whereas heating caused the opposite change^55^. In our case, the thermal side effect cannot be isolated, thus the results show the combined effect of these factors. It is important to note that wet-chemistry methods (alkaline and piranha) do not induce such changes in crystal composition, the PVDF membrane retains its original, predominant γ form. (However, the effect of polymorphs is typically not significant for the performance of membrane processes in practice.)

**Fig. 5:**
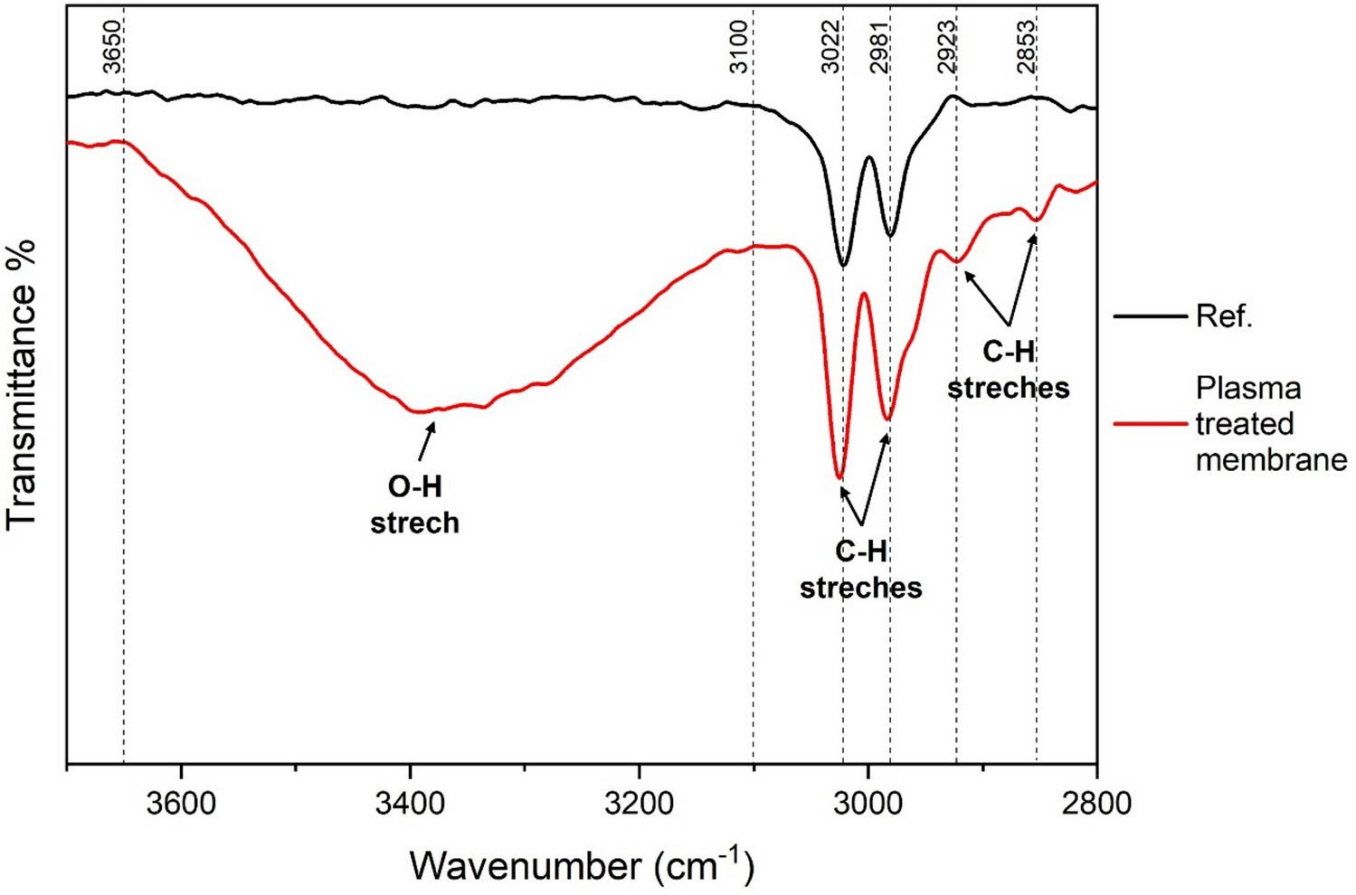
Changes in the IR spectrum of plasma treated (distance = 40 mm; time = 1.0 s). PVDF membrane ($\overline{\nu }$ = 2800–3700 cm^−1^).

Figure 5 shows the IR spectra of plasma treated (using 40 mm distance and 1 s time) and pristine PVDF membrane (standard highly hydrophobic membrane, as a reference) from 2800 cm^−1^ to 3700 cm^−1^.

The peaks at 3022 and 2981 cm^−1^ are assigned to the asymmetric and symmetric stretching of *-CH*_*2*_*–* groups of PVDF, respectively. These peaks are found in both the treated and untreated samples. On the other hand, activated membranes also have a broad peak from 3100 to 3650 cm^−1^, which indicates hydroxyl groups on the membrane surface. In addition, two new peaks appear at 2853 and 2923 cm^−1^, which can be assigned to *–CH*_*2*_*–* bonds with an altered chemical environment. These shifts can be attributed to the substitution of fluorine atoms by hydroxyl groups, and are similar to the spectral changes, which take place using piranha treatment^37^.

#### NMR spectroscopic measurements in solution phase

**Fig. 6:**
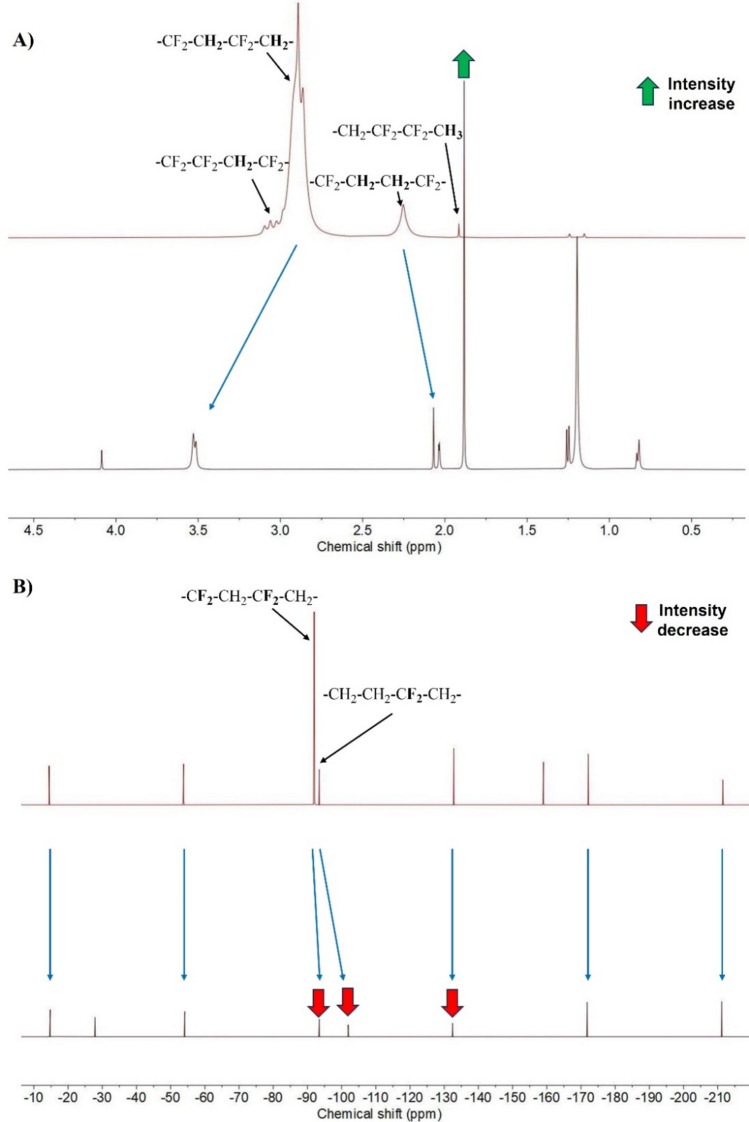
^1^H-NMR (**A**) and ^19^F-NMR (**B**) spectra of pristine and plasma-treated PVDF membranes.

Structural changes due to plasma treatment were also investigated with ^1^H-and ^19^F-NMR spectroscopies (Fig. 6 A, B).

Reference PVDF peaks were characterized based on reported studies^56^. Plasma treatment shifted the peak with the highest intensity at 2.89 ppm to 3.52 ppm, whereas its character remained unchanged. In addition, a new peak appeared at 4.09 ppm. These changes indicate that the environment of the methylene groups altered as the fluorine atoms were substituted by hydroxyl groups. Besides, new peaks appeared in the lower chemical shift region (0.50–1.50) and the intensity of the peak of terminal methyl groups (1.88 ppm) increased, which suggests that chain breakage occurred due to plasma activation. Accordingly, in the ^19^F-NMR spectrum, the intensities of the peaks at − 91.95, − 93.45 and − 132.80 ppm significantly decreased, while the peak at − 159.03 ppm disappeared after the plasma treatment, also demonstrating successful substitution of fluorine atoms. In addition, a new peak appeared (− 27.88 ppm) and minor shifts occurred in the remaining fluorine signals due to plasma modification, as the microenvironment of the remaining fluorine atoms changed because of the substitution by the hydroxyl groups.

**Fig. 7:**
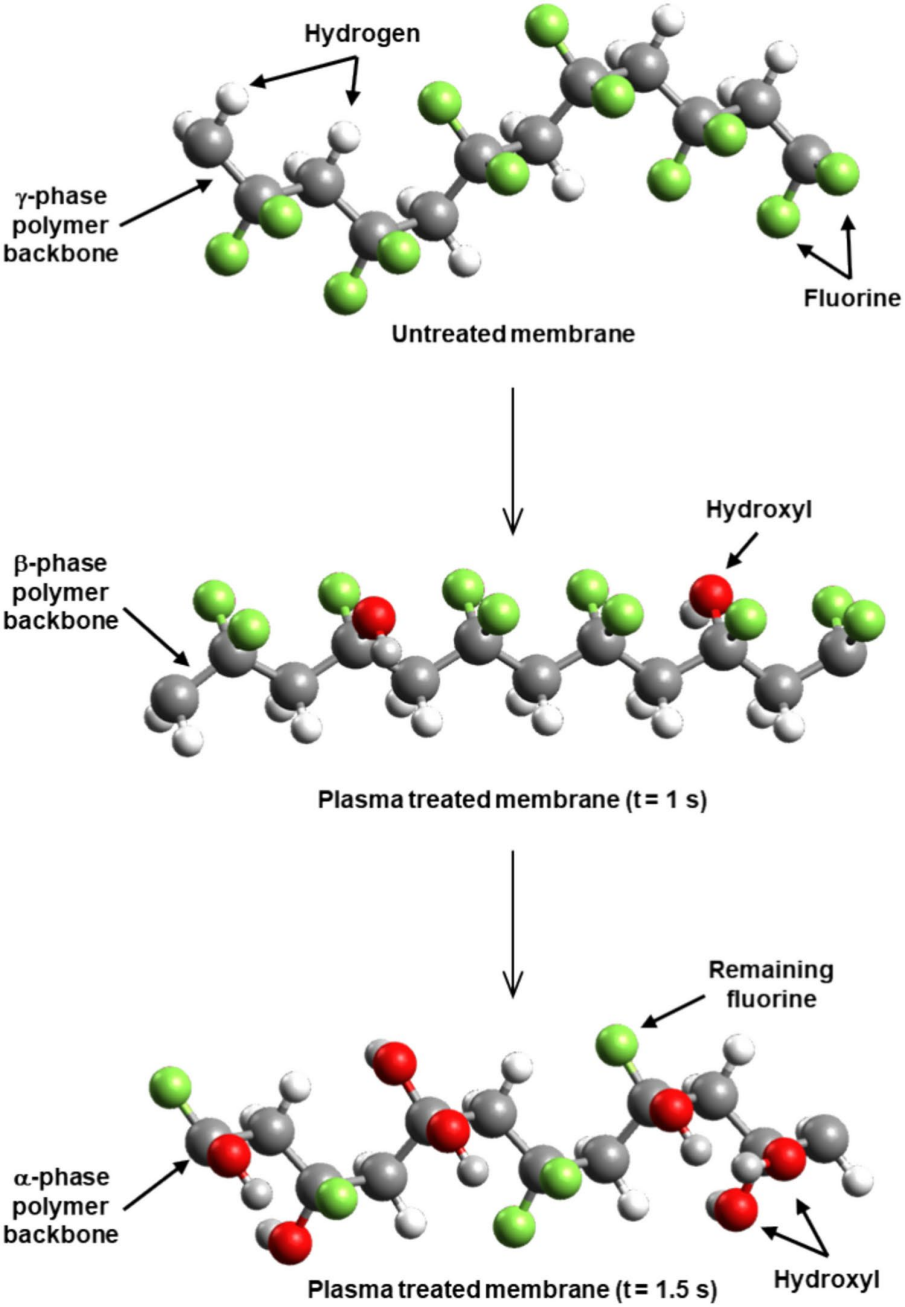
Schematic structure of the activated PVDF polymer chain and the reaction pathway during plasma treatment.

The schematic structure of the activated PVDF polymer chain and the reaction pathway are shown in Fig. 7.

The results show that the degree of substitution of polar groups and the morphology of the PVDF polymer can be influenced by the duration of plasma treatment. IR measurements show that the grafted polar groups on the polymer surface are mainly hydroxyl groups, whereas the quantity of carbonyl functional groups is negligible.

#### SEM measurements

**Fig. 8:**
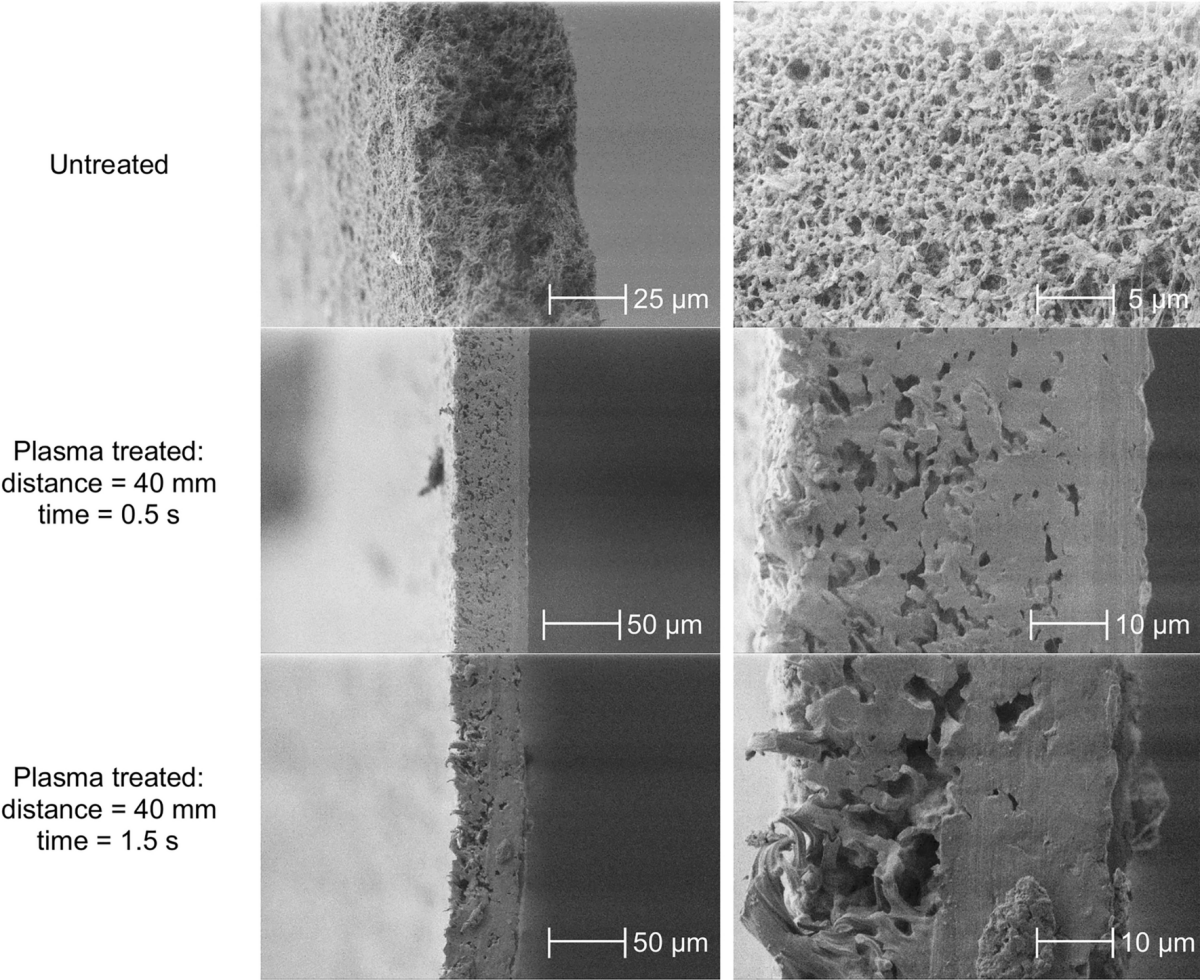
Cross-sectional SEM views of the PVDF membranes.

The cross-section of the membrane and the morphology of the activated surface were examined by scanning electron microscopic (SEM) measurements. SEM records of the cross-section of untreated and treated membranes are shown in Fig. 8 in different magnifications.

The pristine membrane has a thickness of 50 µm, an average pore diameter of 0.45 µm, and it has a fibrous structure as demonstrated by the SEM images of Fig. 8. After a treatment of 0.5 s, the morphology of the membrane has slightly changed, fibers are likely to be partially melted by the thermal effect, resulting in an enhanced pore size distribution and fewer pores. From this perspective, the pores in the upper 10 µm layer appear to have completely merged and practically disappeared. Extending the time of the treatment to 1.5 s, these effects increased, obtaining larger pore size and less pores. The melted and merged range of membrane is wider, covering almost half of the entire cross-section (~ 25 µm).

**Fig. 9:**
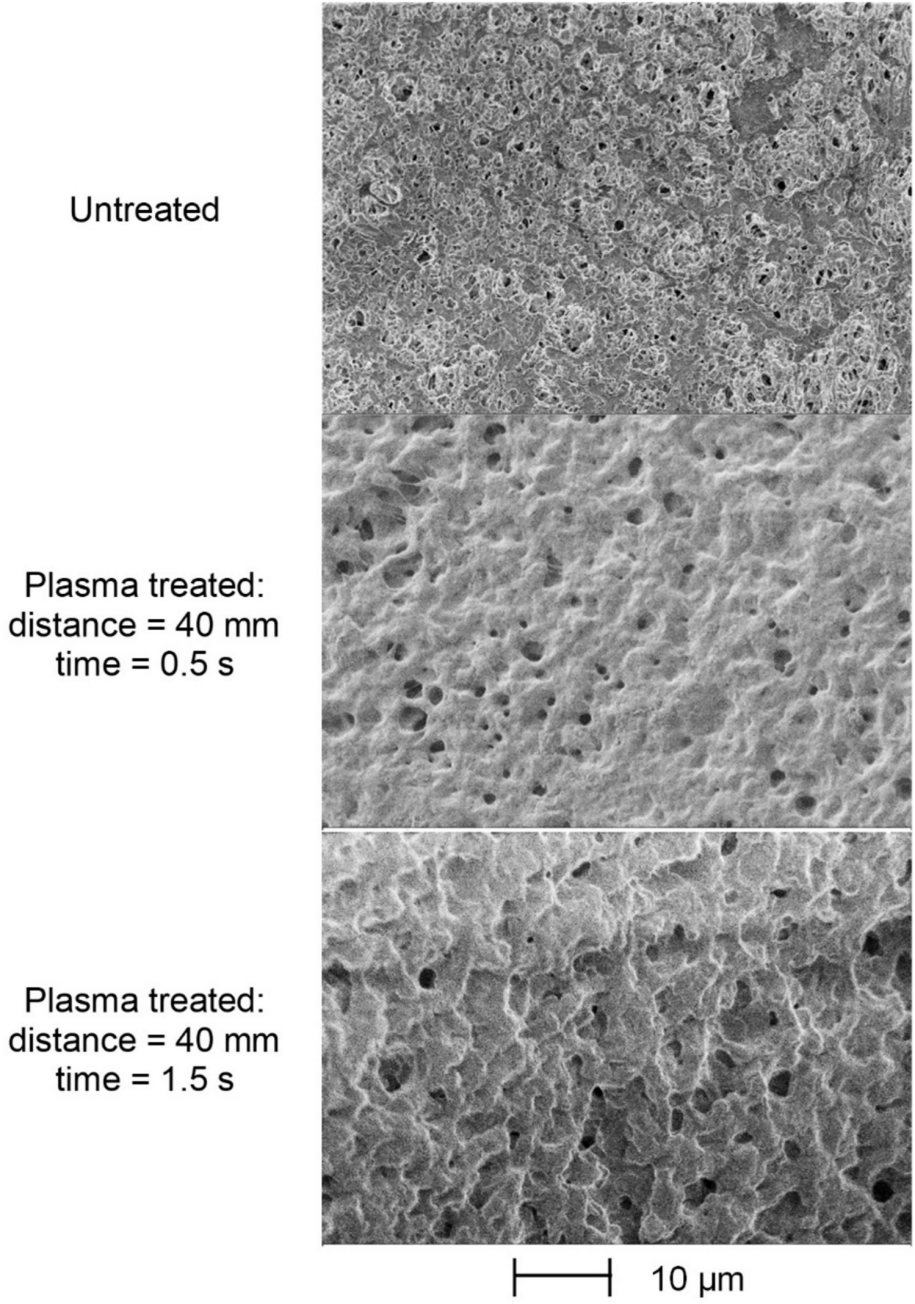
SEM records of the treated surface of PVDF membranes.

Contrary to previous experience, the surficial SEM views (Fig. 9) clearly prove that the pores still exist on the modified surface.

The apparent paradox can be bridged by the fact that during sample preparation, the cutting surface could be slightly deformed, leading to fewer pores being visible in the cross-sectional images. On the other hand, the effect of the increase in treatment time derived from the cross-sectional pictures (increase in pore size and decrease in pore number) is also consistently visible in the surficial images.

**Fig. 10:**
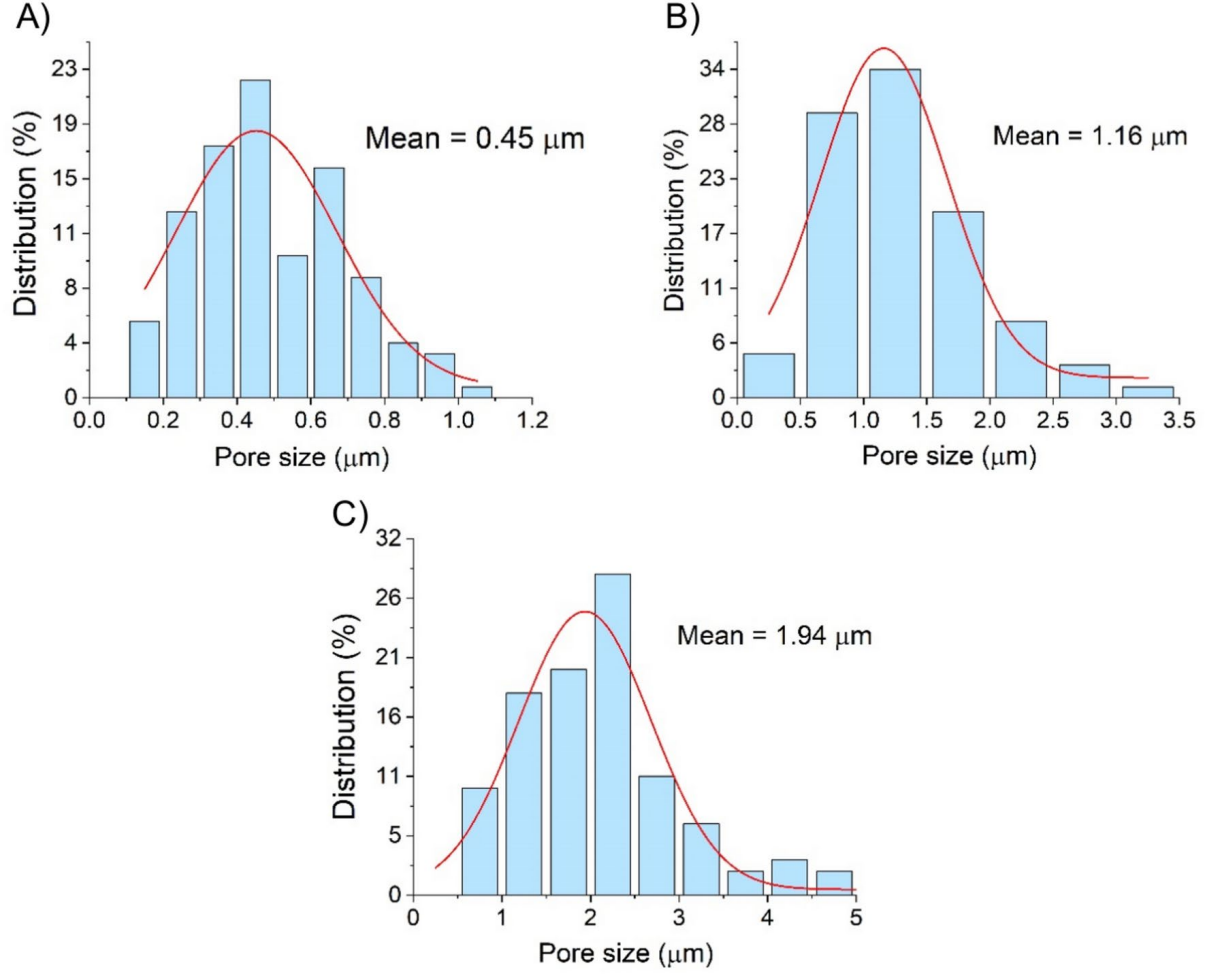
Pore size distribution of untreated (**A**) and plasma treated (**B** 40 mm distance and 0.5 s treatment time;** C** 40 mm distance and 1.5 s treatment time) membranes.

In order to further investigate the membrane morphology, pore size distribution was determined for the untreated and plasma treated polymers based on the cross-sectional SEM images by using image processing method. The pore size distribution diagrams are depicted in Fig. 10.

The pores of the PVDF membrane are partially fused by the thermal effect of the plasma treatment. The diagrams show that this effect also increases with longer treatment time. Initially, the untreated membrane has an average pore diameter of 0.45 µm, which increases to 1.16 µm and then to 1.94 µm as the treatment time progresses.

### Membrane permeability studies

**Fig. 11:**
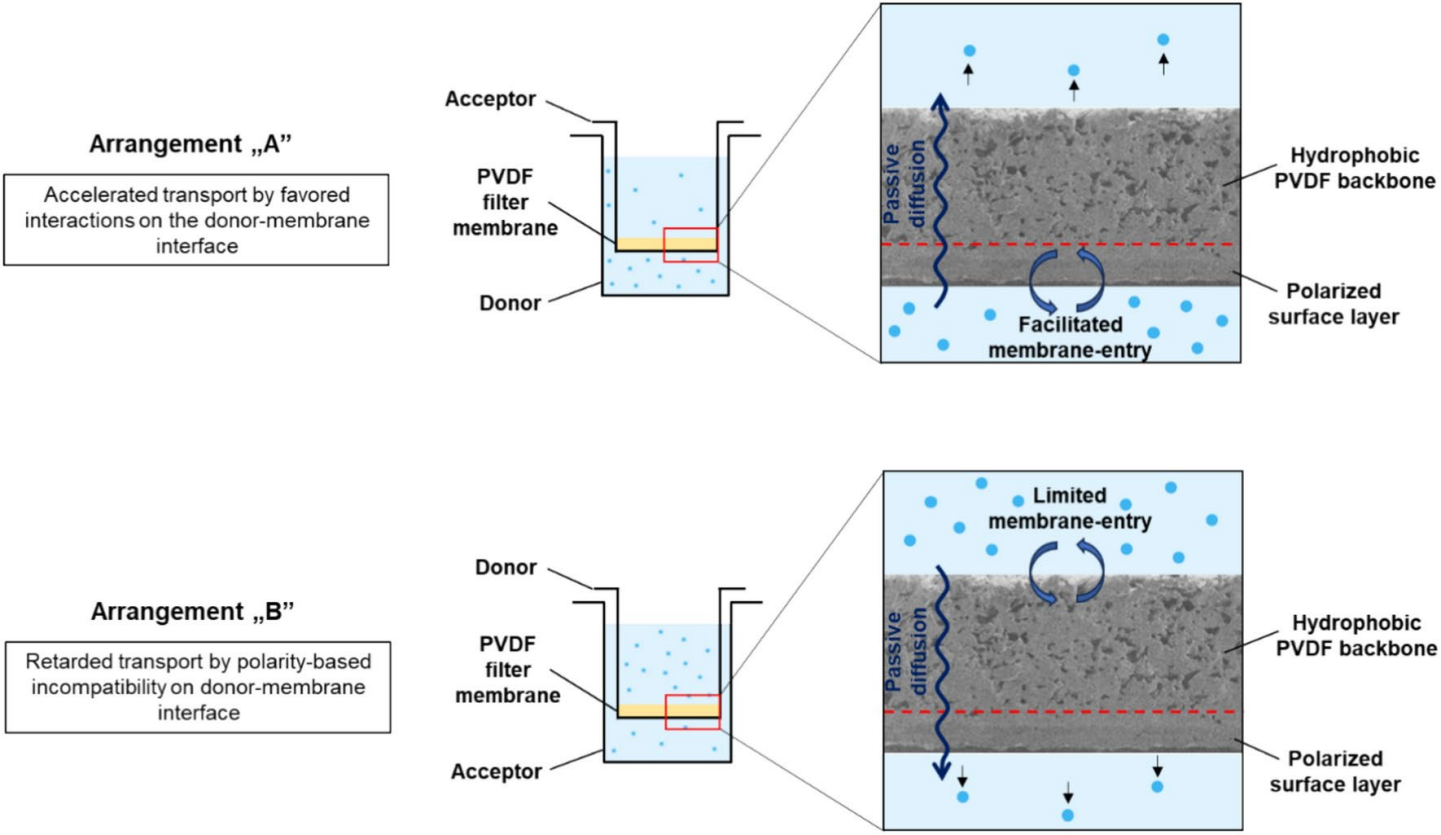
Sandwich-plate system to study the effect of plasma treatment on membrane permeability with two arrangements (A and B).

In order to investigate the possible effect of porosity change on the applicability of membranes, permeability tests were performed with pristine and plasma-treated ones. The membrane permeation of four model compounds was investigated by using a PAMPA-like sandwich assay-system consisting of two aqueous phases separated by the corresponding filter membrane. In order to increase the generalizability of the results, the pH of the donor phase (aqueous solution of the model compound) was adjusted to set different ionization states (molecular charges) of the substances. This concept paves the way to the simultaneous study of polarity-based intermolecular interactions in practice. The model system applied is shown in Fig. 11.

The model compounds applied and their permeability-related main properties are summarized in Table 2, and detailed information on the experimental setup is reported in the Experimental Section.

**Table 2:** Ionization state and lipophilicity of the model compounds applied in membrane permeability studies.

Model compound	pH^1^	Ionization state^2^	log*D*^2^
Mandelic acid (MA)	8.0	negative	− 2.5
*rac*-(1-Naphthyl)ethylamine (NEA)	6.0	positive	− 0.4
Glycine (GLY)	6.0	zwitterionic	− 3.4
5-Methoxybenzene-1,3-diol (ANIS)	7.0	neutral	+ 1.2

^1^The pH of the aqueous donor phase was adjusted by using HCl or NaOH solution.

^2^The ionization state was estimated by software predictions, e.g. lipophilicity at a given pH (ChemAxon, log*D* Plugin). Detailed information on the ionization states of the model compounds can be found in the Supplementary Material.

**Fig. 12:**
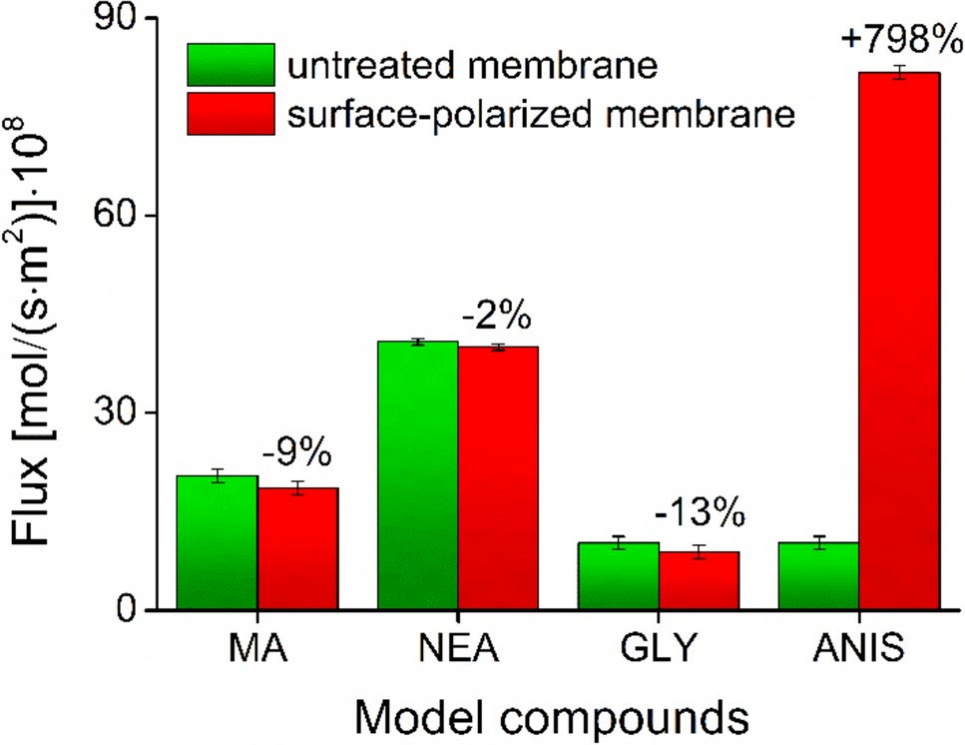
Fluxes of passive membrane transports through untreated and plasma-polarized filter plates by using a sandwich-plate system with arrangement ‘A’ (also see Fig. 11).

As can be seen, all four possible ionization states were investigated. The results for untreated and polarized membranes are compared in Fig. 12.

The results show that a slight reduction in the flux of − 9, − 2 and − 13% was observed in the case of the ionized forms of MA, NEA and GLY model compounds, respectively, when polarized membranes were used in experimental arrangement ‘A’. This was due to the introduced polar groups (mainly *OH*), which caused enhanced retardment by forming second-order interactions in contact with the polarized region of the membrane. The retardment was stronger for compounds containing a negatively charged center (MA and GLY). This is attributed to the possibility for *H*-bond formation between the carbonyl-*O* of the model compound and the surface-*OH* groups of the polymer. In the case of NEA, only ion–dipole interaction can take place, which resulted in a milder influence on permeation. The case of the neutral model compound (ANIS) clearly indicates that the limiting step is the membrane-entry in the donor side-membrane interface. In the absence of polarized regions, neutral species tend to remain in the aqueous phase, despite theoretically showing the highest membrane-affinity. The high hydrophobicity of untreated membrane hinders the material transfer to the acceptor side when only the concentration gradient as a passive driving force governs the process. Upon plasma treatment, the increased polarity of the surface allowed the neutral compound to enter the membrane, even in the absence of forming relatively strong *H*-bonds or ion–dipole interactions. After entering the membrane, the most apolar species can reach the receiver phase faster, resulting in drastically increased permeation after the limiting step was overcome by surface polarization.

When studies were performed in the opposite direction (see experimental arrangement ‘B’ in Fig. 11), this relationship was not observed, so only arrangement ‘A’ had an influence on the permeability of the pristine membranes. Under identical device settings, changing the direction of the material transfer did not solve the entry problem of the neutral species, thus the flux did not increase significantly. Consequently, much smaller changes (statistically not significant) were observed even for the other model compounds (MA, NEA and GLY). Permeability results for experimental arrangement ‘B’ can be found in Supplementary Material. As the plasma treatment left the bulk phase (and also the opposite side) of the membrane unchanged and the rate-limiting step of the material transfer is the donor phase-membrane interfacial transport, there were no significant differences between the results obtained by using arrangement ‘B’ and those of by using the untreated pristine membranes.

In summary, membrane permeability studies proved the presumption that only the porosity of the upper surface was slightly changed during the plasma treatment, and also revealed the importance of the plate arrangements and the role of polarity-based interfacial effects in membrane throughput. (In terms of the latter effects, a similar phenomenon also takes place between lipid membranes and ionized pharmaceutical agents in biomembrane permeability-predicting assays, i.e., the surface ion-pairing (SIP) hypothesis of *Avdeef*^57,58^.) The observed behavior of the treated plates is particularly advantageous, because the treatment provided covalent modification without drastically retarding the membrane passage. Thus, the initially apolar (hydrophobic) and chemically inert membrane matrix can be used without deteriorated properties in practice, as its bulk phase preserved its original features after the treatment. In this way, a favorable dual-function (sufficient surface-wettability and water-penetration, and lack of polar groups and undesired retardment during membrane passage through the bulk phase) was obtained.

### Functionalization of the activated PVDF membranes

**Fig. 13:**
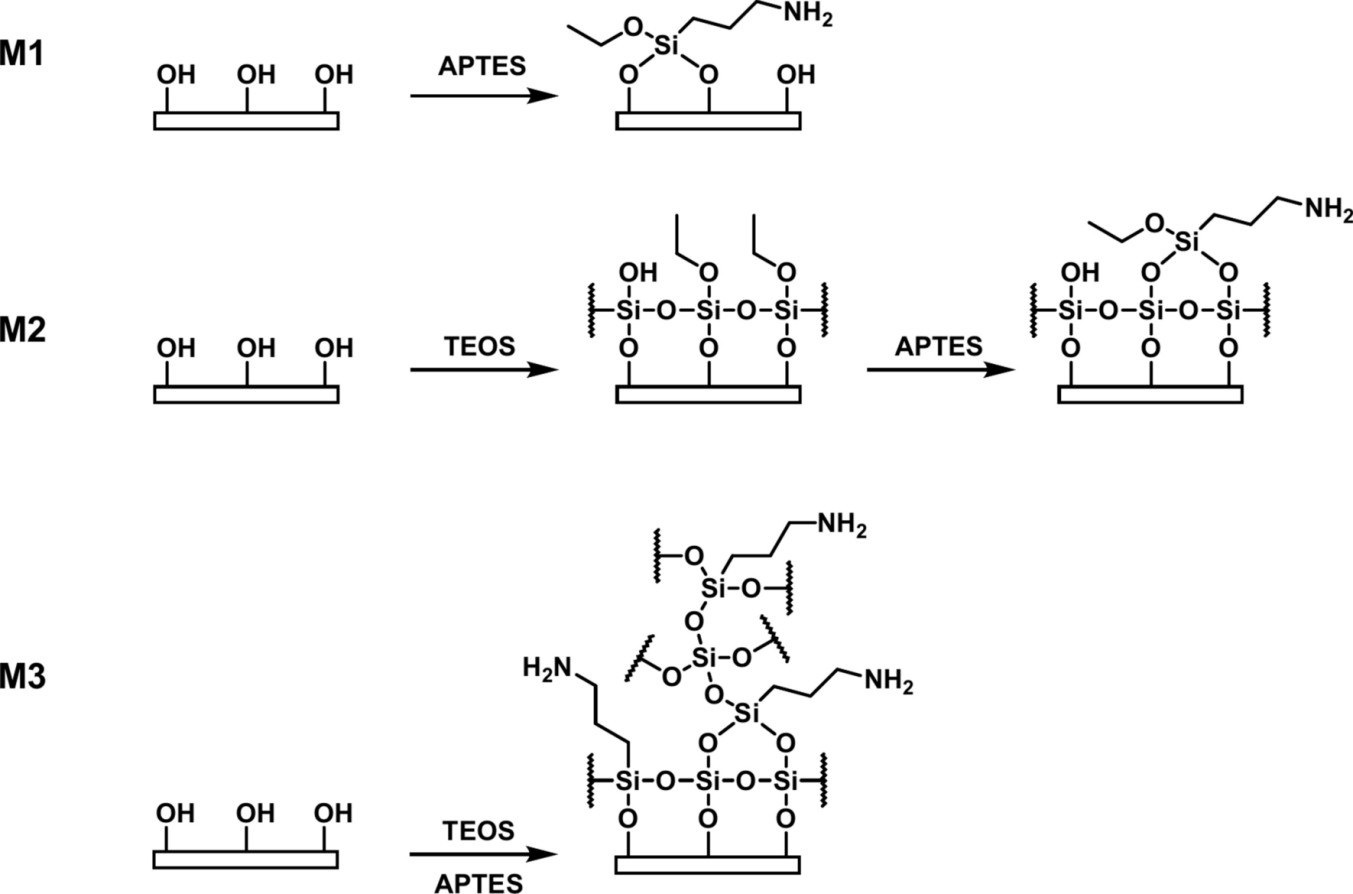
Schematic representation of the three procedures (M1-M3) to introduce linkers with amino group onto the surface of activated polymer.

The plasma-activated membranes can be further functionalized to provide the covalent immobilization of compounds creating linkers with desired functional compounds. Hydroxyl groups formed by plasma treatment allow functional linkers to be covalently attached to the surface. In order to investigate the effectiveness of grafting, a versatile linker with an amino group (possibilities for derivatization by *N*-alkylation, amide formation, reductive amination, ionic bonds, etc.) was introduced by covalently bound (3-aminopropyl)triethoxysilane (APTES) to the membrane surface by three different methods, and the structural changes were examined by ATR-FTIR and NMR spectroscopies, and the extent of loading was determined by UV spectroscopy. The functionalization procedures applied can be seen in Fig. 13.

**Fig. 14:**
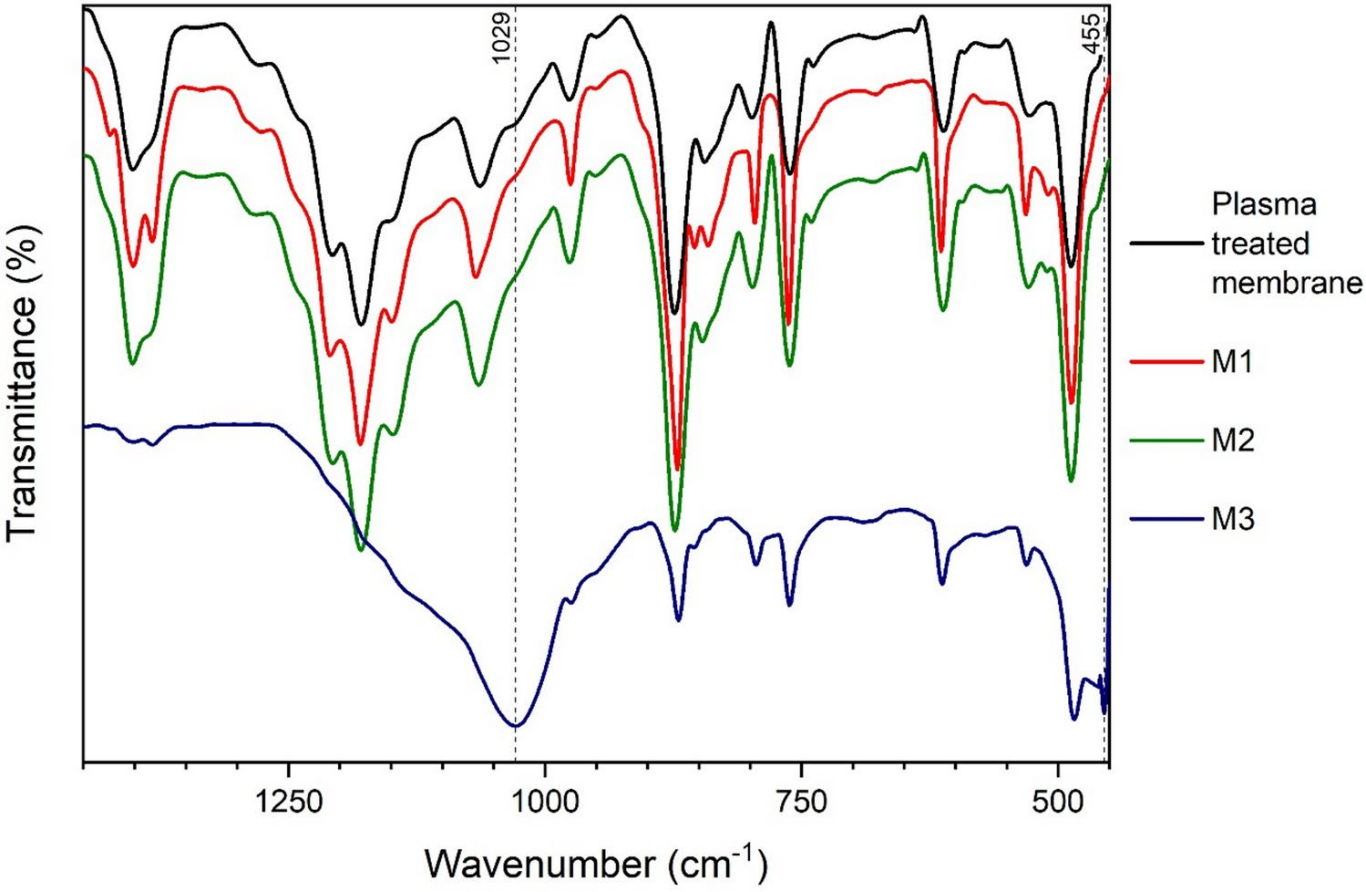
IR spectra of PVDF membranes functionalized by different techniques (M1-M3) ($\overline{\nu }$ = 450–1450 cm^−1^).

For the first method, APTES alone was used directly on the activated surface (M1), for the second method, APTES treatment was applied after a silica-coating by the *in-situ* condensation of tetraethyl orthosilicate (M2), and for the third method, TEOS and APTES were mixed and applied together as a functionalizing solution (M3). The detailed procedures are described in the Experimental Section. Figure 14 shows the IR spectra of treated PVDF membranes obtained by the three different functionalization techniques (M1-M3).

It can be seen that the spectra obtained by methods M1 and M2 do not differ from the spectrum of the original, plasma activated membrane in this range. It means that these functionalization procedures do not affect the conformation. However, it is important to note that small changes in the structure are probably not visible because the whole PVDF polymer layer is much thicker than that of the modified surface one, and the PVDF characteristics suppress those of the APTES and TEOS even in ATR-mode.

In contrast, there is a significant difference in the spectrum of the membrane obtained by the method M3. At 1029 cm^−1^ *Si–O–Si* vibration can be found. This peak is relatively broad, resulting from the overlap of several slightly different *Si–O–Si* vibration peaks. It is typical when the siloxane chain is long and complex. In addition, at 455 cm^−1^, the wavenumber of the *Si–O* rocking vibration also appears in the spectrum. Other peaks are characteristic for PVDF polymer (e.g. 873; 1065; 1179; and 1402 cm^−1^), but they appear with much lower intensity than in the other cases, the vibrations related to *Si–O* bonds are predominant. It can be concluded that in the penetration depth of the IR beam, the detected silica layer is thicker than the PVDF polymer backbone. This measurement result is in line with the apparent experience that the simultaneous additions of APTES and TEOS (procedure M3) result in the formation of a white layer on the membrane and white clusters in the functionalizing solution. As this thick surface layer would obviously impair the permeability of the membrane, it was not investigated further.

**Fig. 15:**
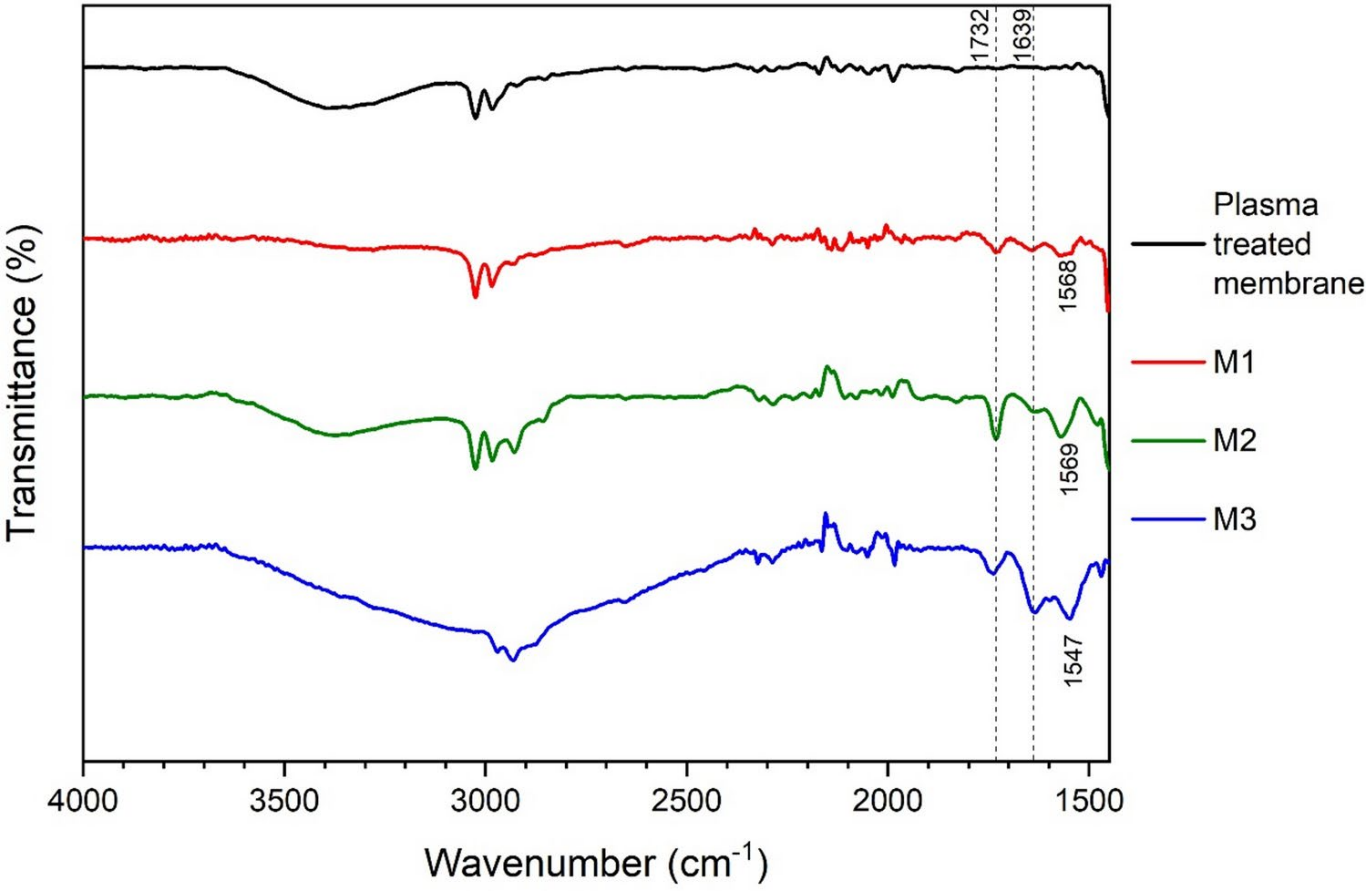
IR spectra of PVDF membranes functionalized by different techniques (M1-M3) ($\overline{\nu }$ = 1450–4000 cm^−^^1^).

The higher wavenumber region of the IR spectra is shown in Fig. 15.

In the case of methods M1 and M2, a broad peak around 3400 cm^−1^ is visible, which can be an overlap of several peaks. This can be the vibrations of unreacted *–OH* groups and the symmetric and asymmetric stretches of the amino groups on the surface. Moreover, it can also be the vibrations of free hydroxyl groups at the *Si* atoms as well as the –*OH* stretches of adsorbed water. The intensity is higher in the case of method M2, where there is a 1–10 µm thick silica layer between the membrane surface and the bound APTES, which indicates the presence of more free hydroxyl groups. The additional silica-layer applying the procedure M2 does not cause an increased thickness of the membrane (according to SEM analysis) and most of the coating material is deposited in the pores. (Information on the characterization of the silica layer in the case of method M2 can be found in Supplementary Material). There is also a change in *C–H* vibrations between 2810 and 3100 cm^−1^, reflecting substitutions in the carbon chain region due to functionalization. In the case of method M3, this region looks completely different, which also demonstrates the subdominant characteristics of the PVDF membrane compared to the silica layer formed on the surface by this method. In all three cases, new peaks are observed in the range from 1500 to 1750 cm^−1^ compared to the plasma treated reference, which can be assigned to the scissor vibration of *NH*_*2*_ and to symmetric and asymmetric *NH*_*3*_^+^ deformation modes^59^.

**Fig. 16.**
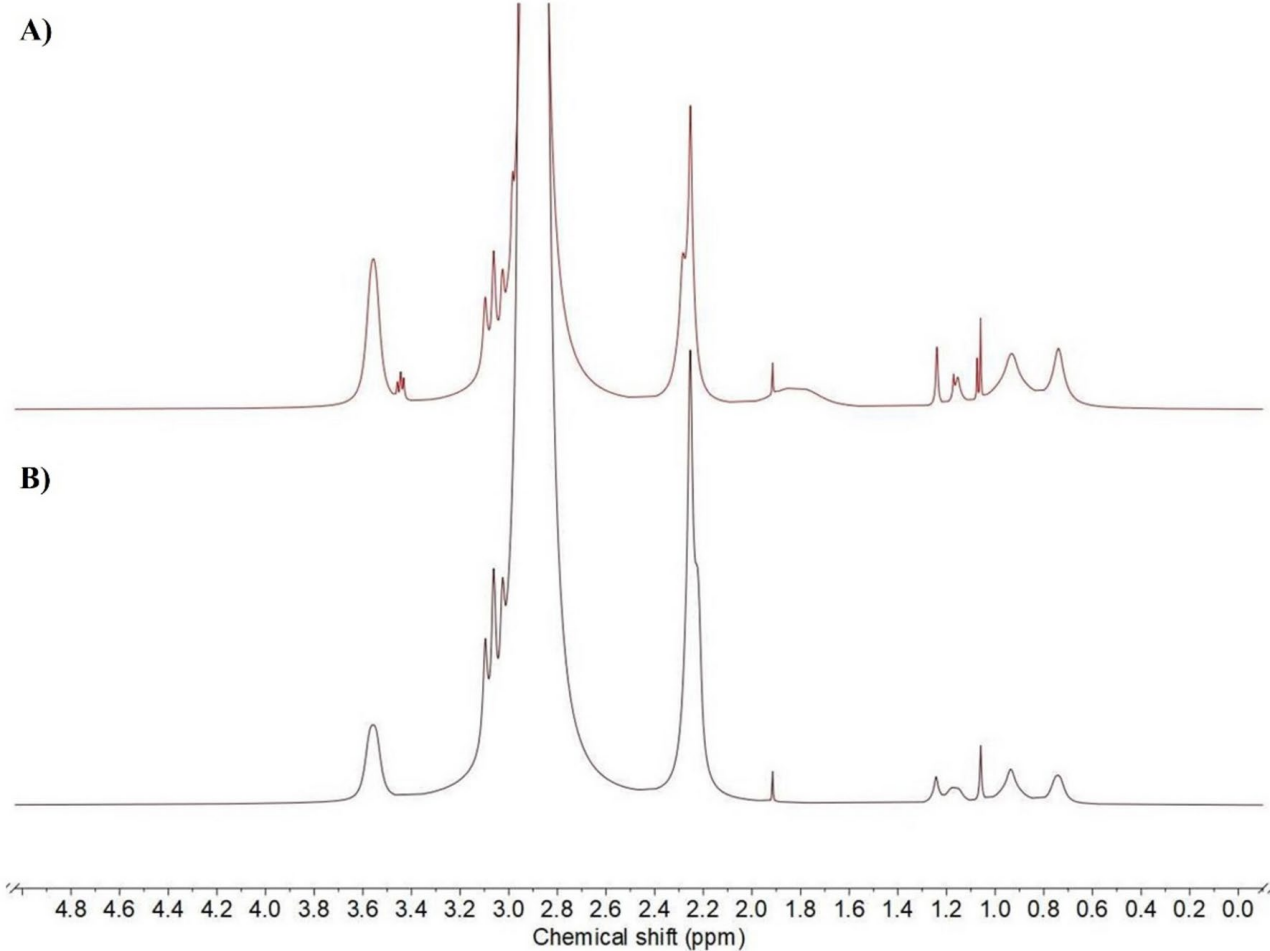
^1^H-NMR spectra of functionalized membranes obtained by methods M1 (**A**) and M2 (**B**).

The membranes functionalized by the methods M1 and M2 were also investigated by ^1^H-NMR spectroscopy. The spectra are shown in Fig. 16.

Both spectra contain characteristic peaks of the pristine PVDF, such as 3.06; 2.89; 2.25; 1.92 ppm and those of the activated membrane, e.g. 3.56 ppm. This means that the plasma does not modify the whole PVDF sheet, but only its top layer. In the lower region, new peaks appear from 0.7 to 1.3 ppm, which can be attributed to the introduced methylene groups of the APTES molecule. As it can be seen from the two spectra, there is no significant difference between the two functionalization procedures (M1 and M2). This also confirms that the silica layer between the PVDF-surface and the APTES-linker is very thin in the case of using the method M2.

The quantity of the bound APTES linker on the surface of the membrane was calculated from the difference between the final (after the reaction) and the initial concentration values of APTES based on their UV absorbance. In the case of method M3, this calculation was not performed because the results would not have been accurate due to the formation of silica clusters. The calculated values are summarized in Table 3.

**Table 3:** Quantity of bound APTES on the pre-activated PVDF membrane surface.

Functionalization method	Reacted quantity (mmol/well)	Physically adsorbed quantity (mmol/well)	Estimated covalently bound quantity (mmol/well)	Estimated covalently bound quantity (m/m% of PVDF/well)
Blank	$2.09\bullet {10}^{-2}$	$2.09\bullet {10}^{-2}$	$0$	0
M1	$2.14\bullet {10}^{-2}$	$2.09\bullet {10}^{-2}$	$5.00\bullet {10}^{-4}$	3.75
M2	$2.13\bullet {10}^{-2}$	$2.09\bullet {10}^{-2}$	$4.00\bullet {10}^{-4}$	3.00

For the blank sample, the functionalization was performed on a membrane, which was not activated by plasma. In this case, the reacted quantity determined by UV spectroscopy is only possible if the model compound was physically adsorbed in the membrane. For methods M1 and M2, the reacted quantity is corrected with this blank value. The remaining part is considered as a minimum amount which is covalently attached to the surface. The membranes were carefully washed after the functionalization (using IPA and distilled water) and no leaching out of the linkers were observed according to the UV spectroscopy. Thus, the linkers were irreversibly attached to the membrane. In summary, there is no significant difference between the extent of labeling with APTES linker by methods M1 and M2. Accordingly, the silica layer between the membrane and the APTES linker was not proved to be essential, the grafting can also be performed by APTES treatment of the pre-activated surface alone. The potential extent of the labeling, which is important for the future general functionalization of membranes, is equal to the covalently bound quantity of the monofunctional linker (4.50 × 10^–4^ mmol/well on average), assuming that each grafted linker molecule acts as active binding site from a modifiability point of view.

## Experimental

### Materials and characterization methods

Starting materials and reagents were purchased from Sigma-Aldrich Corporation (USA, owned by Merck, Darmstadt, Germany) and used without further purification unless otherwise indicated. Non-sterile, 96 well, acrylic filter plates with hydrophobic PVDF membranes (pore size: 0.45 µm) were purchased from Millipore Corporation (owned by Merck, Darmstadt, Germany). For practical reasons, the bottom of the membranes on the conventional acceptor side was treated by plasma. To perform the analytical tests, the membranes were carefully removed from the plate.

For the WCA measurement, 10 µL of distilled water was dropped onto the membrane surface using a microliter syringe (Hamilton Company, Reno, USA). Stable droplets were formed on the membrane surface, neither absorption into the membrane nor dynamic effects were observed during the WCA measurement. Images of the drops were taken with a smart phone in macro mode, WCA was determined using ImageJ software (National Institutes of Health, Bethesda, USA) with a contact angle plug-in based on the ellipse approximation. Ambient temperature and humidity were 25 °C and 60%, respectively.

Infrared spectra were recorded using a PerkinElmer Spectrum Two FTIR (PerkinElmer Inc., Waltham, USA) with a universal ATR accessory head. The fraction of β and γ phases of PVDF relative to the crystalline phases can be calculated from the 837–841 cm^−1^ and 763 cm^−1^ peak intensities using the following equation^54^:

1 $${F}_{\beta +\gamma }=\frac{{A}_{\sim 840}}{\left(\frac{{K}_{\sim 840}}{{K}_{763}}\right)\bullet {A}_{763}+{A}_{\sim 840}}$$

where, ${A}_{\sim 840}$ and ${A}_{763}$ are the absorbances at around 840 cm^−1^ and 763 cm^−1^; ${K}_{\sim 840}$ and ${K}_{763}$ are the absorption coefficients of respective peaks with values of 7.7 × 10^4^ and 6.1 × 10^4^ cm^2^/mol. On the other hand, fraction ratios of the β and γ phases can be distinguished based on the peak-to-valley height ratio between 1275 and 1233 cm^−1^ peaks^53^:

2a $${F}_{\beta }={F}_{\beta +\gamma }\bullet \frac{\Delta {H}_{\beta ; 1275}}{\Delta {H}_{\beta ; 1275}+\Delta {H}_{\gamma ; 1233}}$$

2b $${F}_{\gamma }={F}_{\beta +\gamma }\bullet \frac{\Delta {H}_{\gamma ; 1233}}{\Delta {H}_{\beta ; 1275}+\Delta {H}_{\gamma ; 1233}}$$

where, $\Delta {H}_{\beta ; 1275}$ and $\Delta {H}_{\gamma ; 1233}$ are the height difference between the peak at 1275 cm^−1^ and the nearest valley, and between the peak at 1233 cm^−1^ and the nearest valley. Equations (1); (2a) and (2b) were used to determine the crystalline composition of pristine and plasma treated PVDF membranes.

The ^1^H-NMR and ^19^F-NMR (300 MHz) spectra were recorded on a Bruker 300 Avance spectrometer (Bruker Corporation, Billerica, USA). The membranes for the NMR spectroscopy measurements were dissolved in DMSO-d_6_.

Scanning electron microscopy (SEM) was used for structural analysis of the modified porous membranes by recording secondary and backscattered electron images. The Zeiss LEO 1540XB dual beam system (Carl Zeiss AG, Oberkochen, Germany) was operated with a column vacuum of 2.5–2.6 × 10^–10^, and a system vacuum of 1.6–2.6 × 10^–6^ mbar. Minimized acceleration voltage of 1 kV and low electron beam current (approximately 50 pA) were set to avoid charging of the structures analyzed, given the low electrical conductivity of the polymer membranes. From cross-sectional SEM images, the average diameter of the pores was measured using ImageJ software, assuming the pores to be circular.

### Atmospheric plasma treatment

Plasma treatment was performed using a Plasmatreat FG 5001 Openair-Plasma generator (Plasmatreat GmbH, Steinhagen, Germany). The plasma was generated from compressed air and the pressure was set by a gas reducer equipped with a manometer. Plasma treatment was performed using a Plasmatreat RD1004 rotating plasma head, which was mounted on a frame. The filter plates were attached to a movable support plate and its movement was controlled by a computer. The treatment time was set by moving the support plate at different speeds. The distance between the plasma head and the filter plate varied from 30 to 50 mm. During plasma treatment, 3.5 bar air pressure, 17.5 A electric current and 280 V voltage were fixed.

### Statistical evaluation

The statistical evaluation was performed using STATISTICA 13.4.0.14 software (TIBCO Software Inc., USA). The confidence interval of 95% was fixed in all cases. The studied variables and their levels are listed in Table 4.

**Table 4:** Levels of factors in the experimental design.

Factors	Levels of factors		
	− 1	0	+ 1
Distance (mm)	30	40	50
Time (s)	0.5	1.0	1.5

The applied electric current, voltage and air pressure were kept constant during the study. If the membrane is placed closer than 30 mm to the plasma head, it will melt and rupture immediately, which limits the lower level of the applied distance. All parameters examined had significant effects on the output. The adequacy of the fitted model was checked by *F*-probe, and the model was found to be adequate. For further investigation, the graphical representation of the residues was also checked, such as normality of distribution and the independency of the fitted model. This information can be found in the Supplementary Material.

### UV–Vis spectrophotometric measurements

The UV–Vis spectra were recorded on a UNICAM UV4-100 spectrophotometer controlled by VIZION 3.4 software (ATI UNICAM, Knutsford, UK). Sample concentrations were calculated from absorbance values measured using a calibration curve at a wavelength of 223 nm. The calibration curve was not linear over the whole investigated region (Figure S2 A), so new calibration points were measured at a lower absorbance range to meet *Lambert–Beer* criteria. In this narrowed range, the calibration was adequately linear (*R*^2^ = 0.998; Figure S2 B). Solutions of the samples were diluted to concentrations in this range. The characteristic absorbance curve of the model compound can be found in the Supplementary Material (Figure S3). Spectroscopic data were evaluated and visualized by using OriginPro 2018 software (OriginLab Corporation, Northampton, USA).

### Wet-chemical surface treatments

Conventional chemical activation of the PVDF membrane was performed in two ways. Alkaline activation was performed using a modified literature based method^31^. The membrane was immersed in 200 µL of 5% aqueous NaOH solution for 1 h under an infrared lamp at 40 °C. After the reaction was completed, the membrane was washed three times with distilled water and then dried under an infrared lamp at 40 °C for 24 h.

The activation with acidic piranha solution was performed similarly, based on a modified literature procedure^37^. 750 µL of cc. H_2_SO_4_ (98%) was carefully mixed with 4 mL of distilled water, then 250 µL of cc. H_2_O_2_ (30%) was added to this solution dropwise with continuous stirring. The mixture was cooled to room temperature. To enhance the effectiveness of the treatment, the membrane was pre-wetted with 20 µL of methanol before its immersion in 200 µL of piranha solution for 10 min at room temperature. After the reaction was complete, the membrane was washed with methanol five times, and dried under an infrared lamp at 40 °C for 24 h.

### Covalent functionalization of the pre-activated membranes

In the functionalization step of the activated membranes, three different methods were performed and compared to one another. For procedure M1, only (3-aminopropyl)triethoxysilane (APTES) solution was used. The hydroxylated membrane was immersed in 200 µL of isopropyl alcohol containing 0.15 M of previously added APTES and 0.5 V/V% of H_2_O to promote the hydrolysis of the silane derivative. After 24 h at room temperature, the membrane was washed three times with isopropyl alcohol (IPA) and dried under an infrared lamp at 40 °C for 24 h.

For procedure M2, membranes were treated with 200 µL of 0.15 M TEOS-IPA solution containing 0.5 V/V% of H_2_O for 24 h at room temperature. After that, the membrane was washed three times with IPA, and 0.15 M of APTES-IPA mixture containing 0.5 V/V% of H_2_O was applied for another 24 h. The membrane was then washed again three times with IPA and dried under an infrared lamp at 40 °C for 24 h.

In the third case (procedure M3), APTES and TEOS were used at the same time: the activated membrane was immersed in 200 µL of functionalizing solution containing 0.15–0.15 M of TEOS and APTES and also 1 V/V% of H_2_O in IPA. After 24 h, the membrane was washed three times with IPA and dried under an infrared lamp at 40 °C for 24 h.

### Membrane-permeability studies

The donor phase, regardless of the experimental arrangement, was the solution of the corresponding model compound (250 µL, 0.5 M) in 10% DMSO in water, while there was 150 µL of 10% DMSO-containing distilled water on the acceptor side. The membranes were pre-wetted with 10 µL of DMSO after vacuum suction of IPA (3 × 200 µL). After the sandwich-plate system was set up, a 17-h-incubation was performed at 20 °C. During the experiment, a concentration equilibrium started to establish between the aqueous phases (donor and acceptor) separated by the investigated membranes. The passive transport-governed membrane-passage was interrupted at an early, non-equilibrium state. Flux values were calculated based on the concentration values measured by UV–Vis spectroscopy using the following equation:

3 $$J=\frac{{c}_{a}\bullet {V}_{a}}{t\bullet {A}_{m}},$$

where c_a_ represents the concentration of the acceptor phase at the end point, V_a_ is the volume of the acceptor phase, t is the incubation time and A_m_ is the effective surface area of the filter membrane.

The concentrations of the corresponding model compounds in the acceptor phase were determined according to a previously recorded UV–Vis calibration within a concentration range of 5 × 10^–5^–10^–6^ M (linear concentration-dependent absorbance) at the following wavelengths: 260 nm for MA, 280 nm for NEA, 230 nm for GLY and 260 nm for ANIS.

## Conclusion

We surface-activated and covalently modified widespread commercially available microtiter plates to allow their customizable functionalization in the future, which can be of great benefit for both industrial and academic sectors.

Atmospheric pressure plasma pre-activation was optimized, which surpasses the most common wet-chemistry-based alternatives in polarization efficiency, while does so other-type plasma treatments in potential for industrial implementation, simplicity, rapidity and costs. Subsequent permeability studies revealed the relationships between polarity and charged-driven retardment of the membranes, also referring to transport mechanisms. As a result, favorable dual-functional membranes were obtained (facilitated interfacial water-penetration, while lack of undesired polar interactions during membrane passage through the bulk phase). Finally, three methods were tested and critically compared for functionalizing the pre-activated surface of PVDF membranes, among which reactive amino-linkers with three-*C*–*C*-bond-long molecular spacer units were successfully introduced by covalent bonding directly to the polymer surface. An average of 4.50 × 10^–4^ mmol/well reactive monofunctional binding sites were obtained for further covalent immobilization.

We hope that the present results will provide a useful starting point for microplate-based device development and support the overall progress in high-throughput experimentation.

## Supplementary Material

Statistical validation of the analysis in the experimental design; Effect of long-term storage on membrane hydrophilicity; Different polymorphs of PVDF polymer; UV calibration curve for APTES; UV spectrum of APTES; Ionization states of MA, NEA, GLY, ANIS as a function of pH; log*D*–pH diagrams of MA, NEA, GLY, ANIS; Fluxes of passive membrane transport through untreated and plasma-polarized filter plates by using a sandwich-plate system with arrangement ‘B’; ATR-FTIR based investigation of the silica-layer thickness on the preactivated PVDF membrane surface in functionalization protocol M2.

## Supplementary Information

Supplementary Information 1.

Supplementary Information 2.

## Data Availability

All data generated or analysed during this study are included in this published article and its supplementary information files.
