# Supplementary material for: Atmospheric air plasma pre-activation and customizable covalent functionalization of PVDF-membranes of microtiter filter plates
Source: Sci Rep. 2025 Jan 25;15:3238. doi: 10.1038/s41598-024-85040-5 (PMC11762284; doi:10.1038/s41598-024-85040-5)
Supplement: Supplementary file 1 — Supplementary Information 1. [file 41598_2024_85040_MOESM1_ESM.docx]

**Supplementary Material**

**Covalent functionalization of PVDF-membranes
of microtiter filter plates**

**Bálint Árpád Ádám^[a]^, Sára Spátay^[a,b]^, Bálint Jávor^[a]^, Szabolcs László^[c,d]^,** **Levente Illés^[e]^, Péter Fürjes^[e]^, Tünde Tóth^[a,f]^, Péter Huszthy^[a]^, Ádám Golcs^*[b,g]^**

[a] Department of Organic Chemistry and Technology, Budapest University of Technology and Economics, Szent Gellért tér 4., H-1111 Budapest, Hungary

[b] Department of Pharmaceutical Chemistry, Semmelweis University, Hőgyes Endre u. 9, H-1092 Budapest, Hungary

[c] Department of Inorganic and Analytical Chemistry, Budapest University of Technology and Economics, Szent Gellért tér 4., H-1111 Budapest, Hungary

[d] HUN-REN, Computation-Driven Chemistry Research Group, Műegyetem rkp. 3, H-1111 Budapest, Hungary

[e] HUN-REN Centre for Energy Research, Institute of Technical Physics and Materials Science, Konkoly-Thege Miklós u. 29-33, H-1121 Budapest, Hungary

[f] HUN-REN Centre for Energy Research, Konkoly-Thege Miklós u. 29-33., H-1121 Budapest, Hungary

[g] Center for Pharmacology and Drug Research & Development, Department of Pharmaceutical Chemistry, Semmelweis University, Hőgyes Endre u. 9, H-1092 Budapest, Hungary

*Corresponding author. Tel.: +36 20 622 6164, E-mail address: golcs.adam@semmelweis.hu

**Contents**

**Figure S1.** Statistical validation of the analysis in the experimental design

**Table S1.** α, β, γ, δ, ε and ζ polymorphs of PVDF polymer

**Figure S2.** UV calibration curve for APTES

**Figure S3.** UV spectrum of APTES

**Figure S4.** Ionization states of MA as a function of pH

**Figure S5.** log*D* – pH diagram of MA

**Figure S6.** Ionization states of NEA as a function of pH

**Figure S7.** log*D* – pH diagram of NEA

**Figure S8.** Ionization states of GLY as a function of pH

**Figure S9.** log*D* – pH diagram of GLY

**Figure S10.** Ionization states of ANIS as a function of pH

**Figure S11.** log*D* – pH diagram of ANIS

**Figure S12.** ATR-FTIR based investigation of the silica-layer thickness on the preactivated PVDF membrane surface in functionalization protocol M2


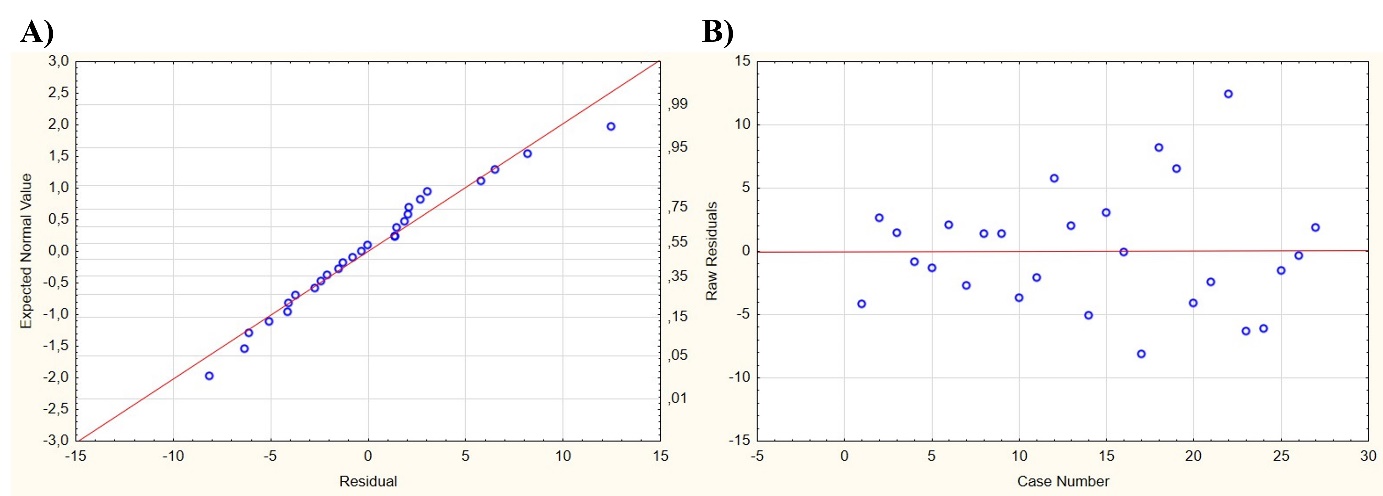


**Figure S1.** Statistical validation of the analysis in the experimental design: **A)** normal probability plot; **B)** raw residuals vs case numbers.

**Table S1.** α, β, γ, δ, ε and ζ polymorphs of PVDF polymer


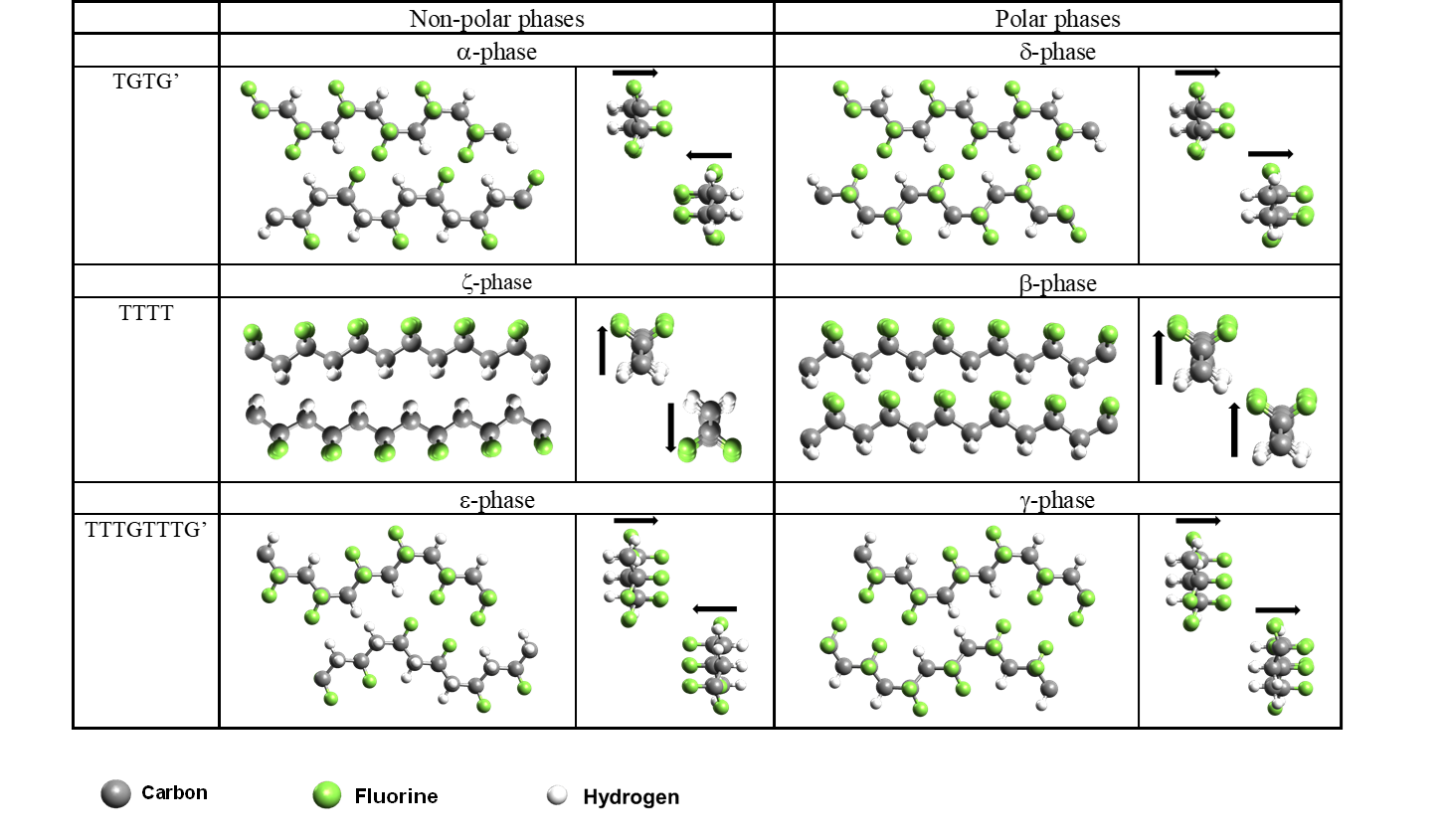


The six different crystal forms can be divided into three groups based on the conformation of the polymer chains (Table S1, rows). In α and δ phases the chains have trans-gauche-trans-gauche (TGTG’) conformation, in ζ and β phases all trans, while in ε and γ phases 3 trans-gauche-3 trans-gauche (TTTGTTTG’). On the other hand, the PVDF has two different types of polymorphs based on the chain orientations (Table S1, columns): in case of α, ζ and ε phases the dipole moments of the chains are antiparallel, making them non-polar, while in the δ, β and γ polymorphs the dipole moments of the polymer chains are aligned, resulting in electroactive, polar phases.


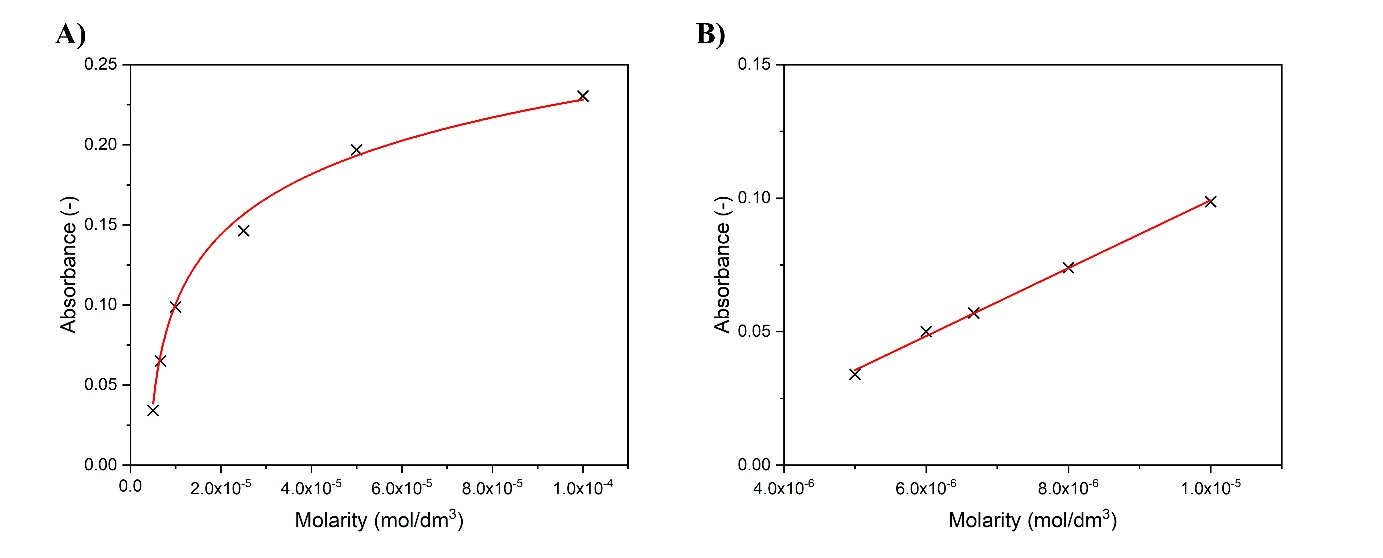


**Figure S2.** UV calibration curve for APTES **A)** from 5×10^-6^ to 10^-4^ M;

**B)** from 5×10^-6^ to 10^-5^


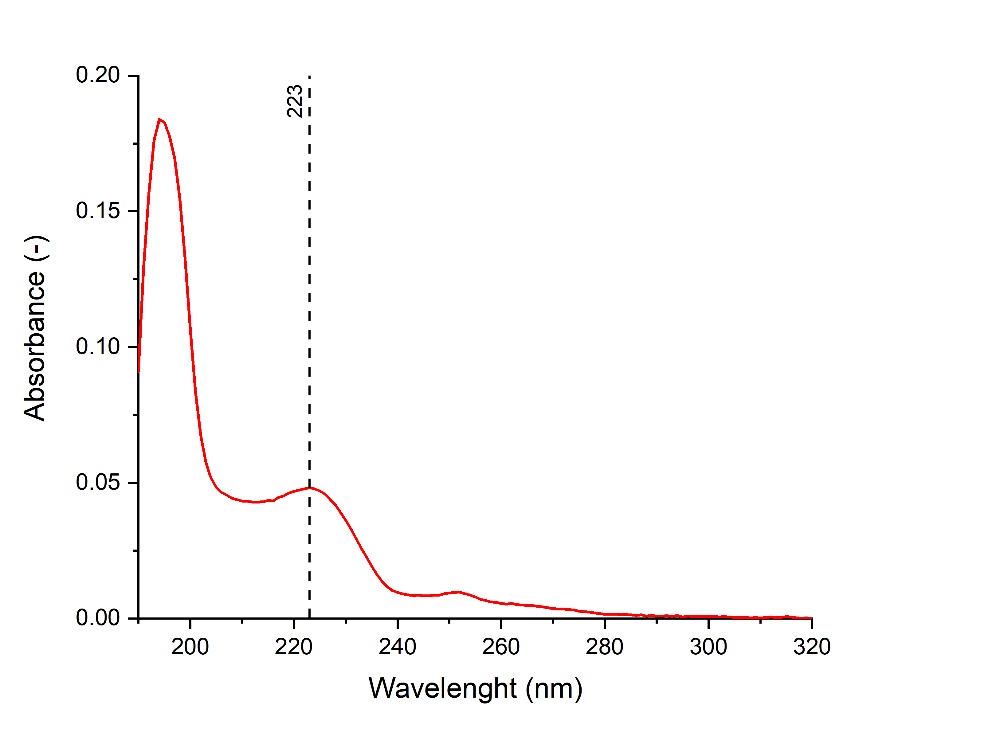


**Figure S3.** UV spectrum of APTES


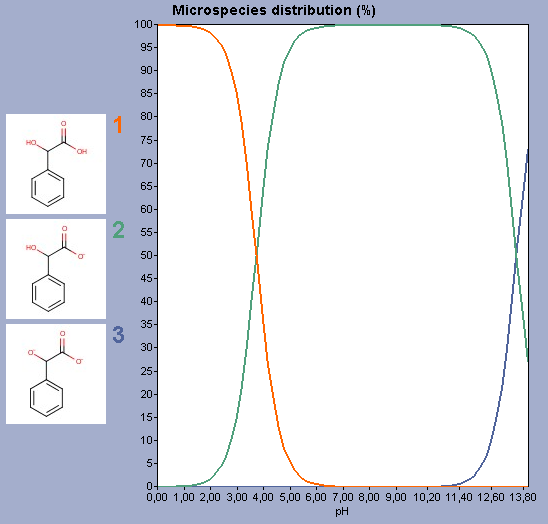


**Figure S4.** Ionization states of MA as a function of pH


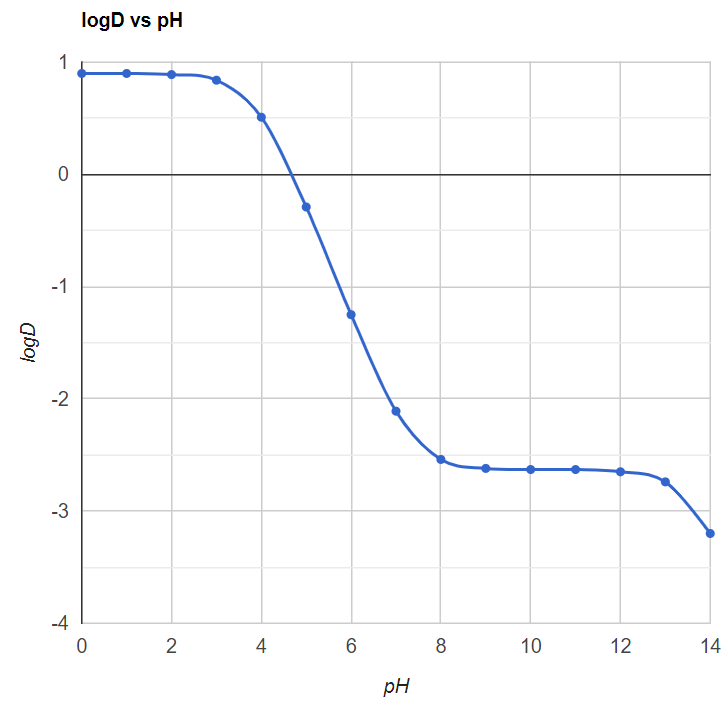


**Figure S5.** log*D* – pH diagram of MA


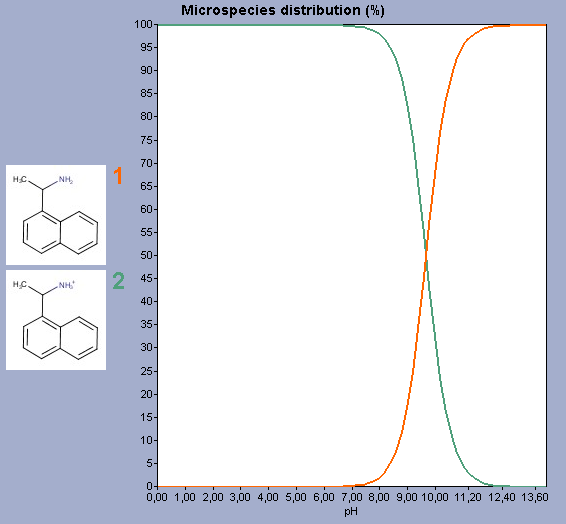


**Figure S6.** Ionization states of NEA as a function of pH


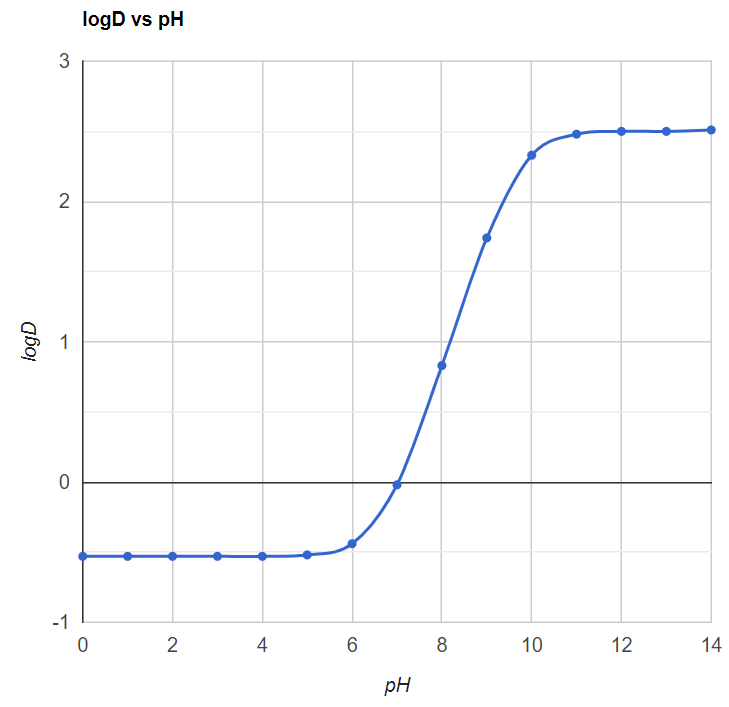


**Figure S7.** log*D* – pH diagram of NEA


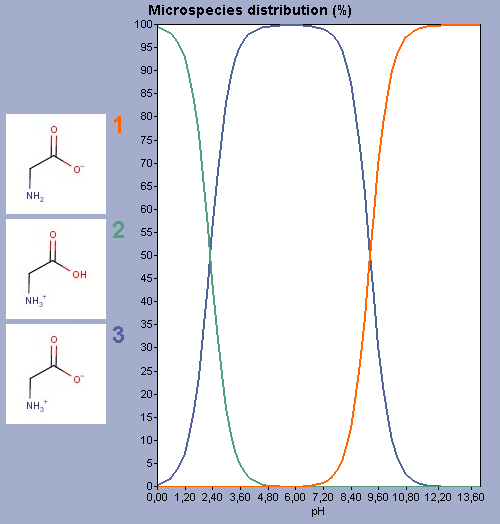


**Figure S8.** Ionization states of GLY as a function of pH


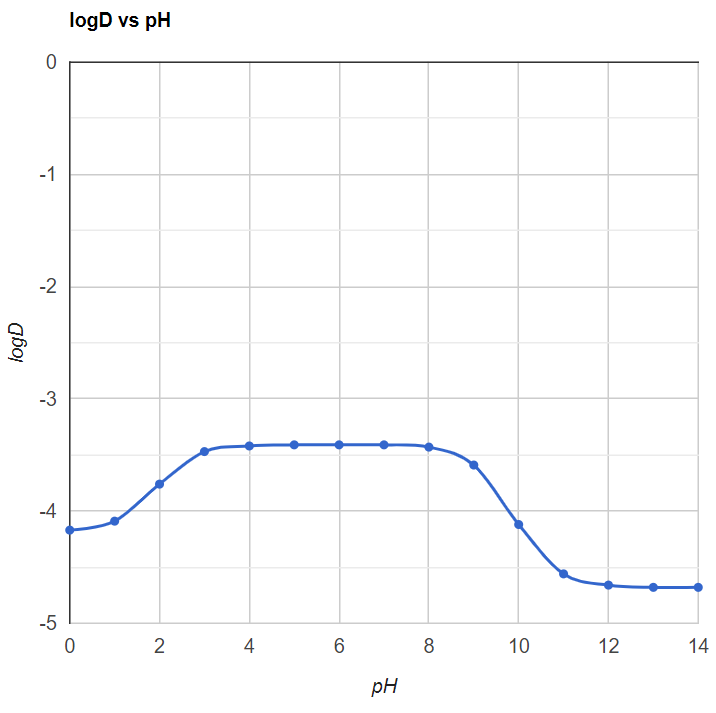


**Figure S9.** log*D* – pH diagram of GLY


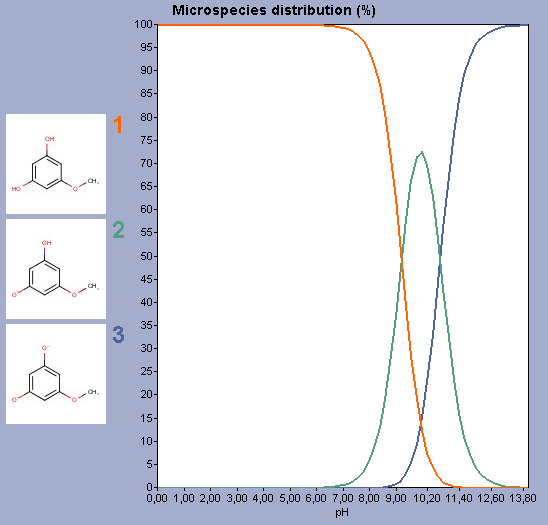


**Figure S10.** Ionization states of ANIS as a function of pH


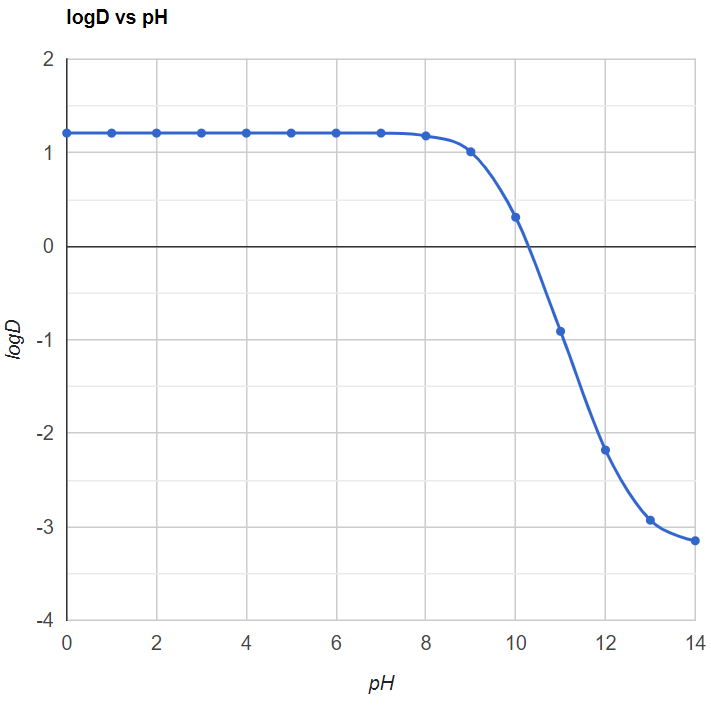


**Figure S11.** log*D* – pH diagram of ANIS





**Figure S12.** ATR-FTIR based investigation of the silica-layer thickness on the preactivated PVDF membrane surface in functionalization protocol M2 (calibration spectra were recorded on the thickness range of 0-100 µm, while the thickness of the formed layer after applying procedure M2 was determined based on the characteristic peak ratios)
