# Supplementary material for: Atmospheric air plasma pre-activation and customizable covalent functionalization of PVDF-membranes of microtiter filter plates
Source: Sci Rep. 2025 Jan 25;15:3238. doi: 10.1038/s41598-024-85040-5 (PMC11762284; doi:10.1038/s41598-024-85040-5)
Supplement: Supplementary file 2 — Supplementary Information 2. [file 41598_2024_85040_MOESM2_ESM.pdf]

Supplementary Material

Atmospheric air plasma pre-activation and customizable covalent functionalization of PVDF-membranes of microtiter filter plates

Bálint Árpád Ádám[a], Sára Spátay[a,b], Bálint Jávor[a], Szabolcs László[c,d], Levente Illés[e], Péter Fürjes[e], Tünde Tóth[a,f], Péter Huszthy[a], Ádám Golcs*[b,g]

[a] Department of Organic Chemistry and Technology, Budapest University of Technology and Economics, Szent Gellért tér 4., H-1111 Budapest, Hungary

[b] Department of Pharmaceutical Chemistry, Semmelweis University, Hőgyes Endre u. 9, H-1092 Budapest, Hungary

[c] Department of Inorganic and Analytical Chemistry, Budapest University of Technology and Economics, Szent Gellért tér 4., H-1111 Budapest, Hungary

[d] HUN-REN, Computation-Driven Chemistry Research Group, Műegyetem rkp. 3, H-1111 Budapest, Hungary

[e] HUN-REN Centre for Energy Research, Institute of Technical Physics and Materials Science, Konkoly-Thege Miklós u. 29-33, H-1121 Budapest, Hungary

[f] HUN-REN Centre for Energy Research, Konkoly-Thege Miklós u. 29-33., H-1121 Budapest, Hungary

[g] Center for Pharmacology and Drug Research & Development, Semmelweis University, Üllői u. 26., H-1085 Budapest, Hungary

*Corresponding author. Tel.: +36 20 622 6164, E-mail address: golcs.adam@semmelweis.hu

Contents

Figure S1. Statistical validation of the analysis in the experimental design

Figure S2. Effect of long-term storage on membrane hydrophilicity

Table S1. α, β, γ, δ, ε and ζ polymorphs of PVDF polymer

Figure S3. UV calibration curve for APTES

Figure S4. UV spectrum of APTES

Figure S5. Ionization states of MA as a function of pH

Figure S6. log*D* – pH diagram of MA

Figure S7. Ionization states of NEA as a function of pH

Figure S8. log*D* – pH diagram of NEA

Figure S9. Ionization states of GLY as a function of pH

Figure S10. log*D* – pH diagram of GLY

Figure S11. Ionization states of ANIS as a function of pH

Figure S12. log*D* – pH diagram of ANIS

Figure S13. Fluxes of passive membrane transports through untreated and plasma-polarized filter plates by using a sandwich-plate system with arrangement 'B'

Figure S14. ATR-FTIR based investigation of the silica-layer thickness on the preactivated PVDF membrane surface in functionalization protocol M2

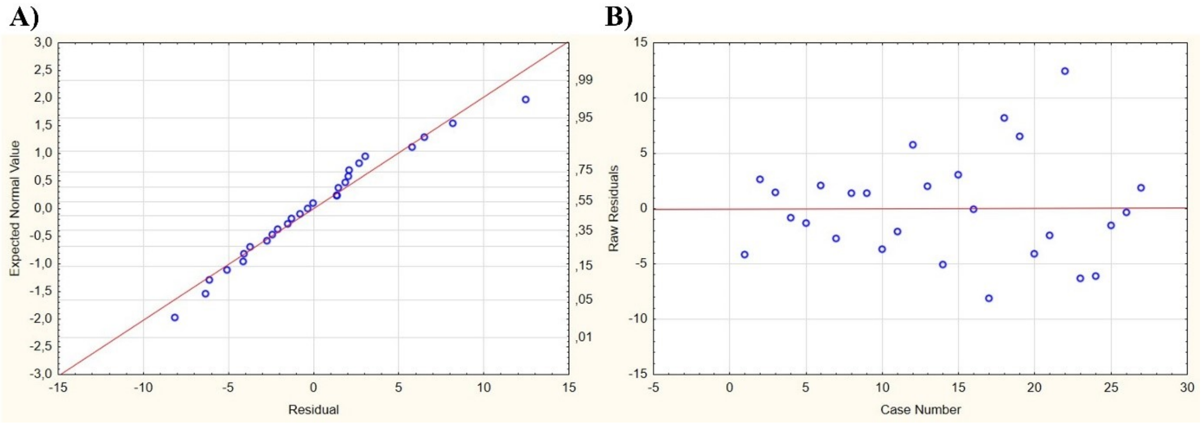

Figure S1. Statistical validation of the analysis in the experimental design: **A)** normal probability plot; **B)** raw residuals vs case numbers.

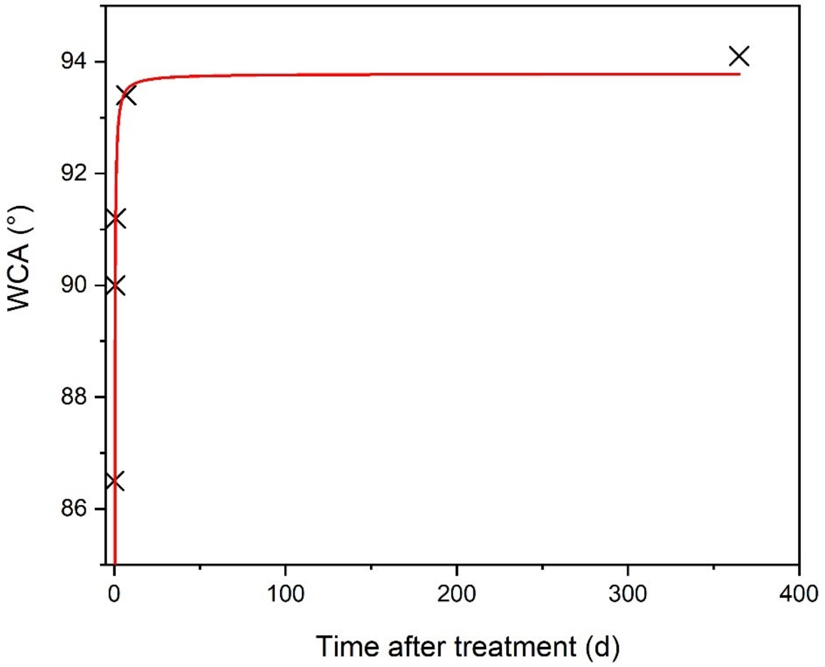

Figure S2. Effect of long-term storage on membrane hydrophilicity

Table S1. α, β, γ, δ, ε and ζ polymorphs of PVDF polymer

	Non-polar phases		Polar phases	
	α-phase		δ-phase	
TGTG’	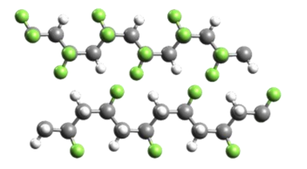	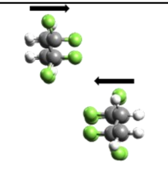	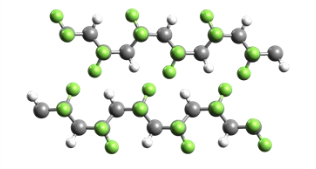	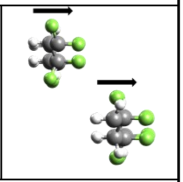
	ζ-phase		β-phase	
TTTT	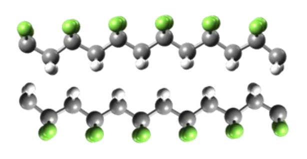	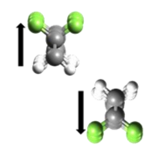	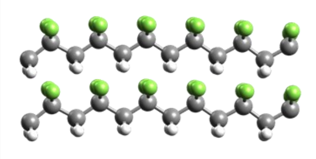	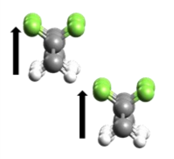
	ε-phase		γ-phase	
TTTGTTTG’	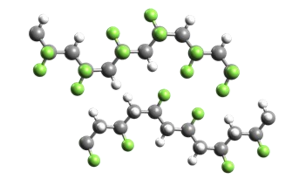	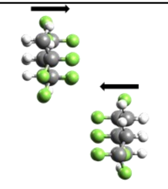	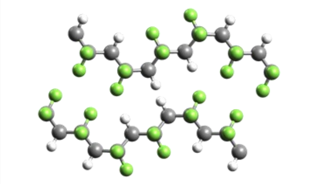	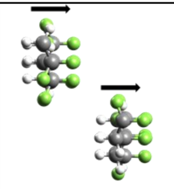

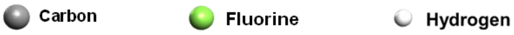

The six different crystal forms can be divided into three groups based on the conformation of the polymer chains (Table S1, rows). In α and δ phases the chains have trans-gauche-trans-gauche (TGTG’) conformation, in ζ and β phases all trans, while in ε and γ phases 3 trans-gauche-3 trans-gauche (TTTGTTTG’). On the other hand, the PVDF has two different types of polymorphs based on the chain orientations (Table S1, columns): in case of α, ζ and ε phases the dipole moments of the chains are antiparallel, making them non-polar, while in the δ, β and γ polymorphs the dipole moments of the polymer chains are aligned, resulting in electroactive, polar phases.

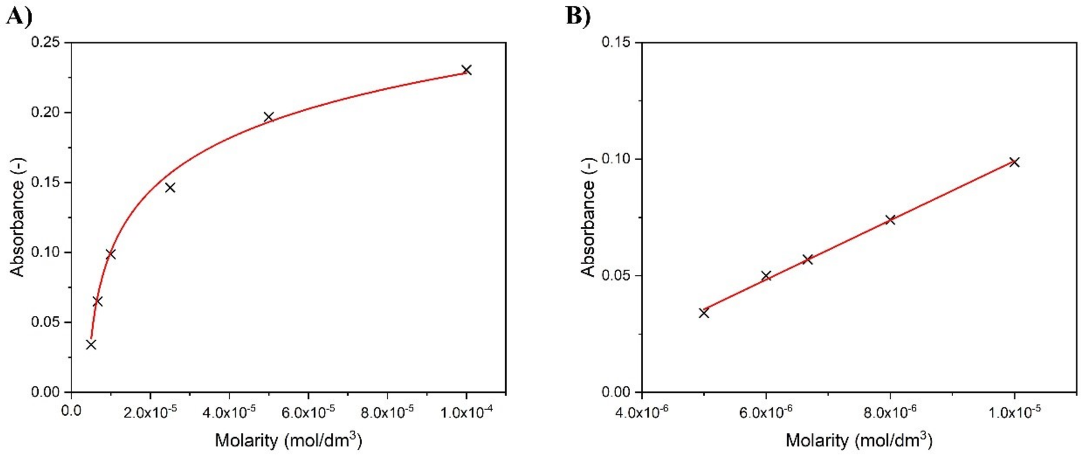

Figure S3. UV calibration curve for APTES **A)** from 5×10^{-6} to 10^{-4} M; **B)** from 5×10^{-6} to 10^{-5}

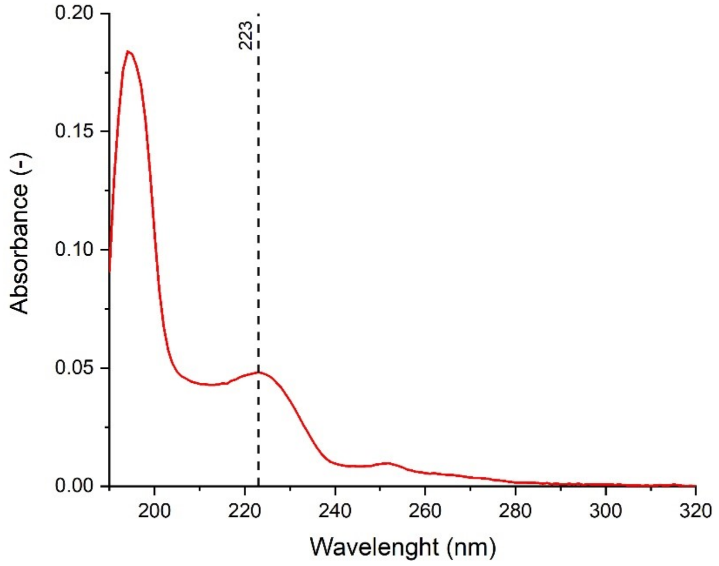

Figure S4. UV spectrum of APTES

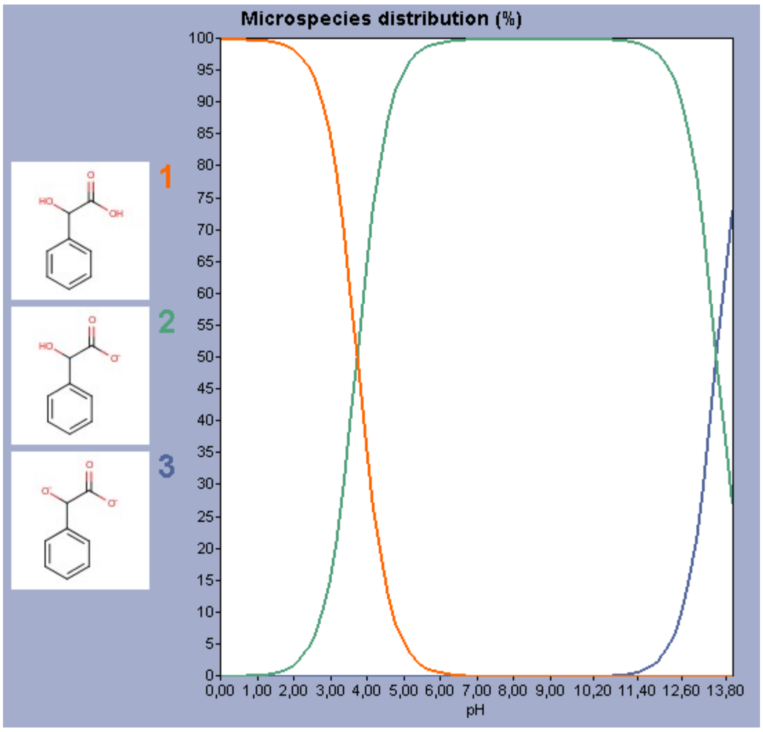

Figure S5. Ionization states of MA as a function of pH

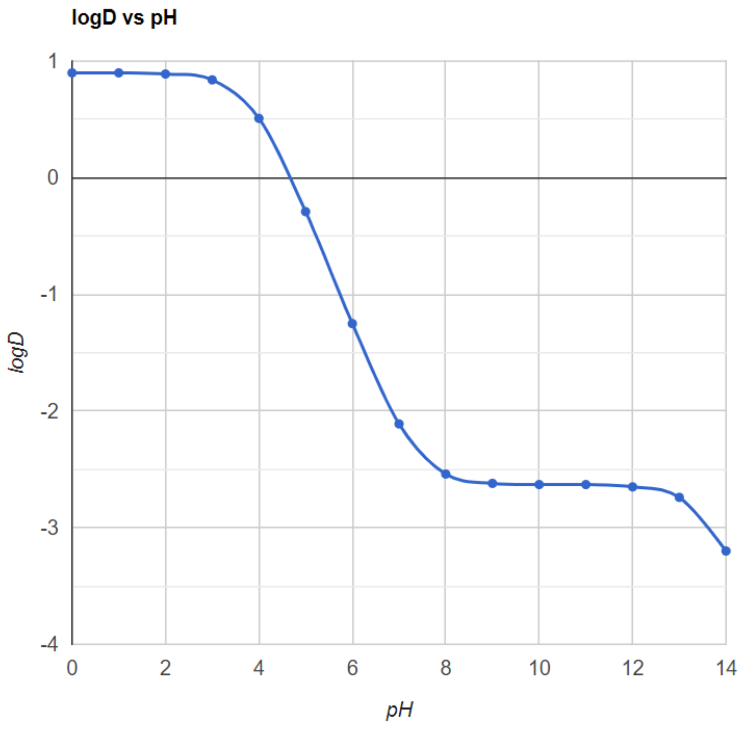

Figure S6. log$D$ – pH diagram of MA

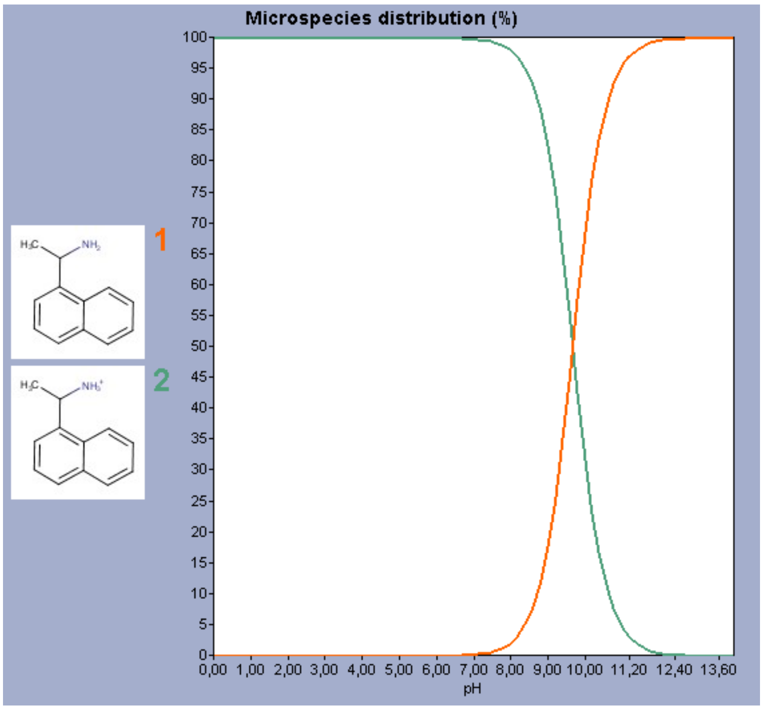

Figure S7. Ionization states of NEA as a function of pH

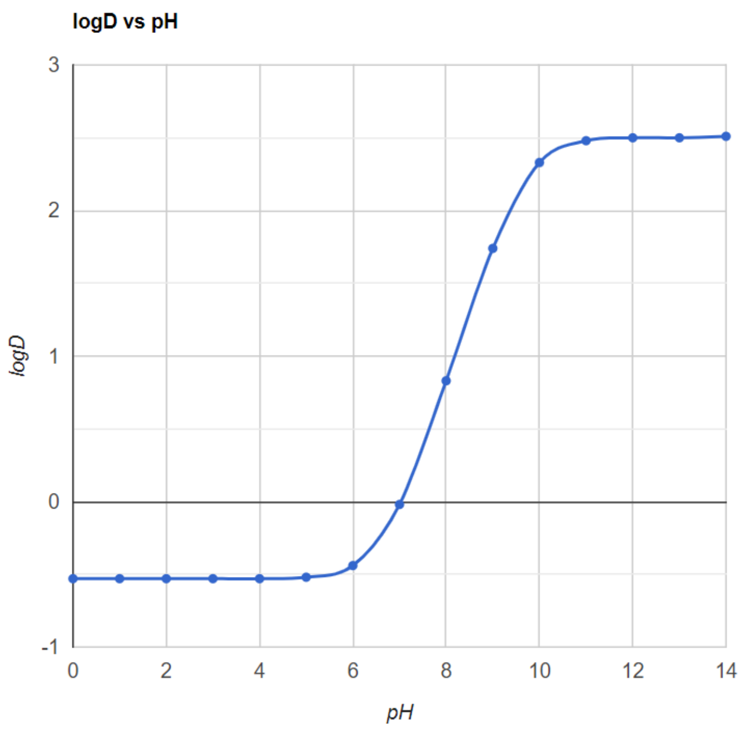

Figure S8. log*D* – pH diagram of NEA

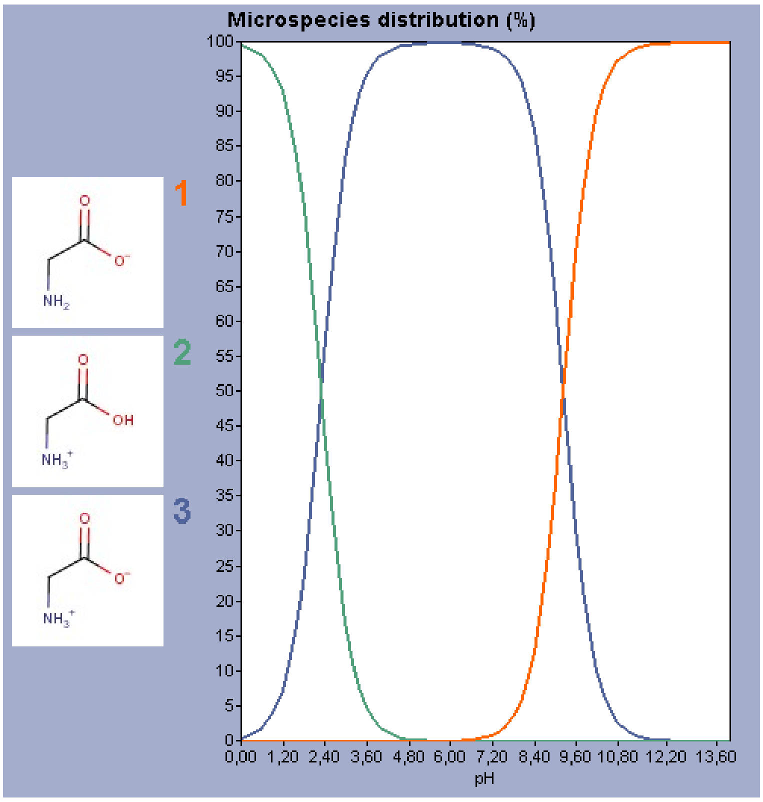

Figure S9. Ionization states of GLY as a function of pH

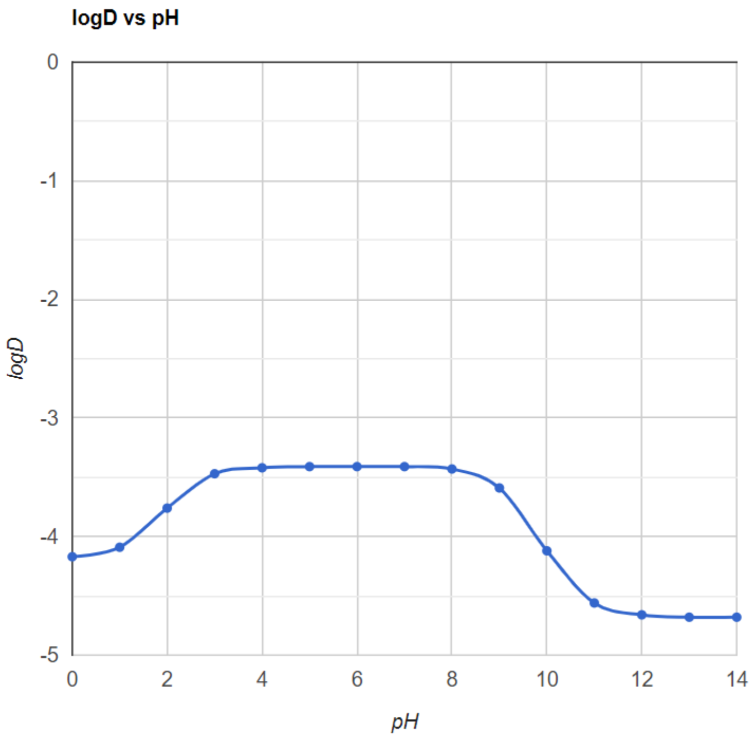

Figure S10. log*D* – pH diagram of GLY

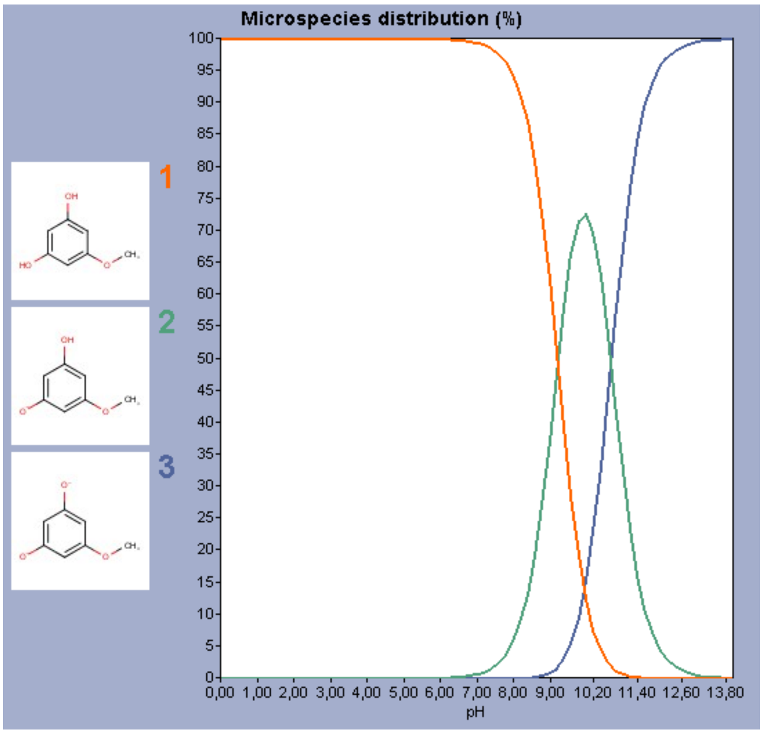

Figure S11. Ionization states of ANIS as a function of pH

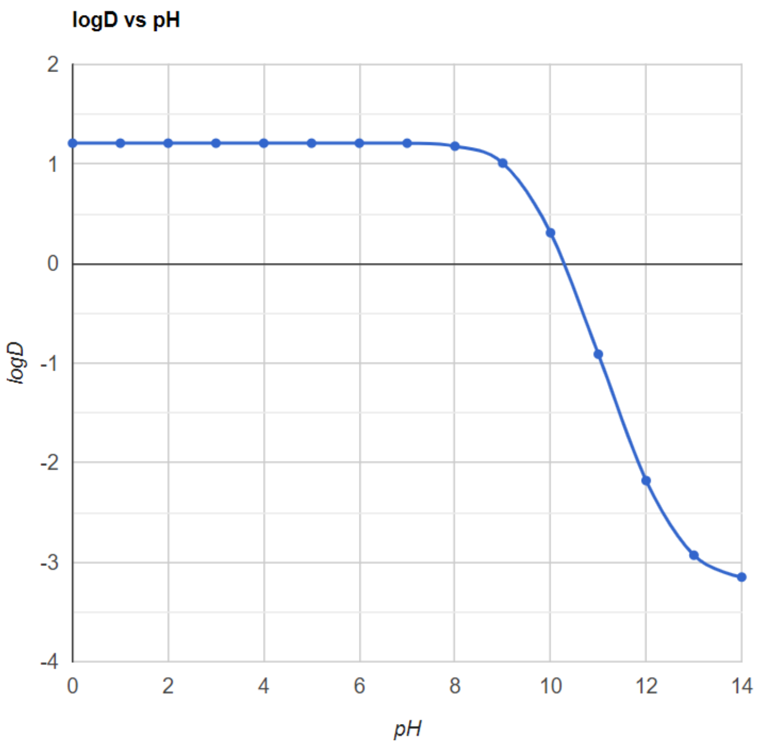

Figure S12. log*D* – pH diagram of ANIS

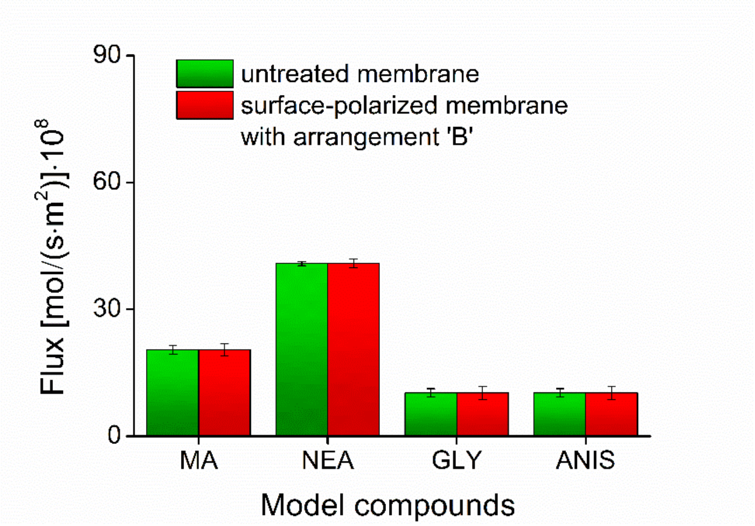

Figure S13. Fluxes of passive membrane transports through untreated and plasma-polarized filter plates by using a sandwich-plate system with arrangement ‘B’

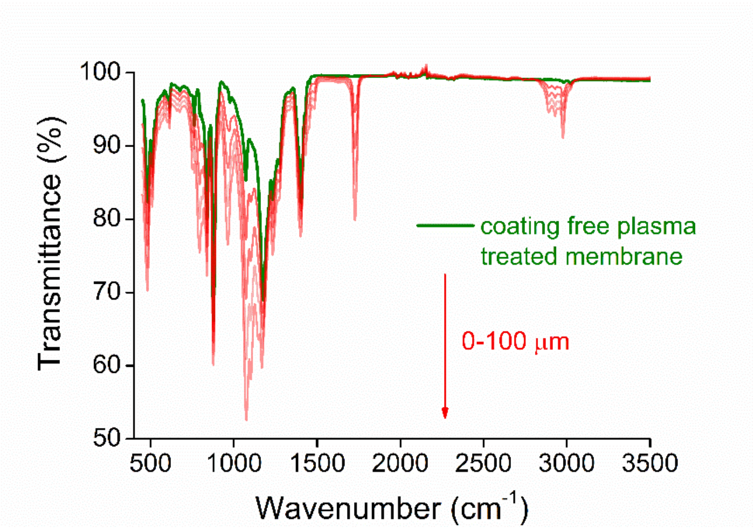

Figure S14. ATR-FTIR based investigation of the silica-layer thickness on the preactivated PVDF membrane surface in functionalization protocol M2 (calibration spectra were recorded on the thickness range of 0-100 µm, while the thickness of the formed layer after applying procedure M2 was determined based on the characteristic peak ratios)
